# Integrated Transcriptomic and Phytohormone Metabolomic Analyses Reveal Distinct Molecular States Associated with PSTVd-Induced Potato Tuber Cracking and Knob-like Protuberances at Tuber Eyes

**DOI:** 10.3390/plants15182842

**Published:** 2026-09-17

**Authors:** Guangyan Li, Wajahat Hussain, Ting Pan, Yingyan Chen, Jiaqi Li, Yunxia Zeng, Yonghong Zhou, Dianqiu Lv

**Affiliations:** 1 College of Agronomy and Biotechnology, Southwest University, Chongqing 400715, China; lihayy616@163.com (G.L.); wajahat7253@yahoo.com (W.H.); 15736270797@163.com (T.P.); 13118924035@163.com (Y.C.); 15136826253@163.com (J.L.); 17341868521@163.com (Y.Z.); 2 Integrative Science Center of Germplasm Creation in Western China (Chongqing) Science City, Southwest University, Chongqing 400715, China; 3 Chongqing Key Laboratory of Potato Biology and Genetic Breeding, Southwest University, Chongqing 400715, China

**Keywords:** potato spindle tuber viroid, tuber cracking, knob-like protuberances at tuber eyes, region-dependent molecular states, transcriptome, phytohormones

## Abstract

Potato spindle tuber viroid (PSTVd) infection can induce tuber cracking and knob-like protuberances at tuber eyes, but the molecular differences between these two localized symptoms remain unclear. In this study, smooth surface regions and normal eye tissues were collected from mock-inoculated tubers of the potato cultivar ‘Kexin 18’, whereas crack regions and regions of knob-like protuberances at tuber eyes were collected from PSTVd-infected tubers. RNA sequencing and targeted phytohormone quantification were combined with weighted gene co-expression network analysis, infection × region interaction analysis, and gene-hormone association analysis. PSTVd infection markedly inhibited plant growth and tuber development, and 2972 genes with significant infection × region interaction effects were identified. Crack regions were characterized primarily by enhanced defense and oxidative stress related transcriptional responses, remodeling of cell wall and surface barrier related processes, and accumulation of several cytokinin (CK) metabolites, salicylic acid/salicylic acid glucoside (SA/SAG), and 12-oxo-phytodienoic acid (OPDA); *CRK2*, *RBOHA*, and *CSLG2* were among the representative candidate genes. Regions of knob-like protuberances at tuber eyes showed pronounced reprogramming of processes related to DNA replication, the cell cycle, chromosome maintenance, and chromatin organization, together with decreases in several CK metabolites, a downward trend in jasmonate-related oxylipins, and an increase in indole-3-butyric acid; *ACL5-like*, *PILS7-like*, *CYCA3;1*, and *MCM4* were identified as candidate genes. Gene-hormone association analysis further revealed distinct molecular association patterns in the two regions. Collectively, the two symptomatic regions exhibited distinct region-dependent transcriptomic and phytohormone metabolic states after symptom development.

## 1. Introduction

Potato (*Solanum tuberosum* L.) is an important food and processing crop. Its tubers, the principal harvested organs, develop through enlargement of the subapical region of underground stolons. Tuber initiation and bulking directly determine yield, whereas quality traits such as dry matter, starch, and reducing-sugar contents influence nutritional and processing quality [1,2]. Potato spindle tuber viroid (PSTVd), the first viroid to be identified, is a non-protein-coding, single-stranded circular RNA with a rod-like secondary structure. It depends on host factors for nuclear replication through an asymmetric rolling-circle mechanism and moves from cell to cell and systemically through plasmodesmata and the phloem [3,4,5]. PSTVd infection can cause plant dwarfing, leaf deformation, and yield loss, as well as small and spindle-shaped tubers, cracking, deep eyes, and knob-like protuberances at tuber eyes. Some infected tubers remain asymptomatic but can still transmit PSTVd as seed tubers, and symptom type and severity are influenced by host genotype, PSTVd strain, and environmental conditions [6,7,8,9].

Previous studies indicate that PSTVd-host interactions involve multiple layers of regulation, including RNA silencing, transcriptional reprogramming, epigenetic regulation, and phytohormone signaling. RNA silencing can restrict PSTVd replication and accumulation [10]. Transcriptomic studies in potato have shown that PSTVd infection broadly affects defense responses, carbohydrate metabolism, hormone signaling, and genes associated with tuber development [11]. PSTVd can also alter alternative splicing of host genes, the trans-regulatory activity of phased secondary small interfering RNAs (phasiRNAs), and immune responses [12], while inducing transcriptional reprogramming of MAPK signaling, reactive oxygen species (ROS)-related defense, photosynthesis, cell wall metabolism, and hormone signaling [13]. Viroid infection may also be accompanied by changes in host DNA methylation [14]. Phytohormone networks connect immune responses with growth and development and coordinate defense and growth through synergistic and antagonistic interactions [15]. PSTVd-induced endogenous hormone and antioxidant responses are organ-specific [16]. Integrated transcriptomic and hormone analyses have shown that jasmonic acid (JA) and its signaling pathway contribute to basal immunity and symptom regulation in PSTVd-infected potato. Inhibition of JA biosynthesis or perception suppresses defense-related gene expression and H_2_O_2_ accumulation, alleviates symptoms, and increases PSTVd accumulation [17]. Overall, these studies indicate that PSTVd infection is associated with coordinated changes in multiple host regulatory processes [18].

Although tuber cracking, deep eyes, and knob-like protuberances at tuber eyes have been reported in PSTVd-infected potato [8,9], most previous studies have examined responses at the whole-plant, leaf, or whole-tuber level. The localized molecular states associated with tuber crack regions and regions of knob-like protuberances at tuber eyes remain poorly characterized. The tuber periderm consists of phellem, phellogen, and phelloderm and serves as a major barrier against water loss and pathogen invasion. Its formation and maturation depend on phellogen activity, cell wall remodeling, and suberin deposition [19,20]. During wound healing, phenylpropanoid metabolism is activated early, followed by increased fatty acid synthesis and modification [21], and transcription factors such as *StMYB24*, *StMYB144*, and *StMYB168* participate in suberin formation [22]. Suberin biosynthesis and assembly also involve fatty acid elongation, glycerol esterification, and cell wall cross-linking [23,24], while transcriptomic analysis of the phellogen has identified regulatory processes associated with periderm formation, cell division, and maturation [25]. Hormone homeostasis also contributes to wound repair: abscisic acid (ABA) promotes wound suberization and barrier restoration [26], whereas cytokinin precursors and metabolites show temporal changes and indole-3-acetic acid (IAA) increases during wound periderm formation; GAs remain below quantifiable levels, suggesting possible roles for CK and IAA in wound periderm development [27]. These findings indicate that disruption of periderm barrier formation, wound repair, and hormone homeostasis may compromise the structural stability of the tuber surface and adjacent tissues and may be associated with PSTVd-induced tuber cracking.

Knob-like protuberances at tuber eyes are associated with altered regulation of local growth and development. Release from tuber dormancy and sprout growth are controlled by complex genetic factors [28] and are accompanied by changes in carbohydrate metabolism and hormone-regulatory networks [29,30]. ABA, GA, CK, auxin, and other phytohormones jointly regulate dormancy maintenance, dormancy release, and sprout growth [29,31,32], and their effects are closely linked to source-sink relationships [30]. However, the relationships among local cell proliferation, metabolic status, and hormone homeostasis during the formation of PSTVd-induced knob-like protuberances at tuber eyes remain unclear. PSTVd infection may therefore generate a localized molecular response associated with these protuberances by reprogramming growth, development, and metabolism in the eye region.

Accordingly, the potato cultivar‘Kexin 18’(K18) was subjected to mock inoculation or inoculation with a severe PSTVd strain. Smooth surface tissue and normal eye tissue were collected from mock-inoculated tubers, and crack-region tissue and tissue from knob-like protuberances at tuber eyes were collected from PSTVd-infected tubers. Transcriptomic and phytohormone metabolomic analyses were integrated with weighted gene co-expression network analysis (WGCNA), analyses of key hormone contents and relative ratios, and gene-hormone correlation network construction. Shared and region-dependent responses were compared to characterize the distinct molecular features associated with PSTVd-induced tuber cracking and knob-like protuberances at tuber eyes.

## 2. Results

### 2.1. PSTVd Infection Inhibits Growth of Potato K18 and Induces Two Distinct Tuber Abnormalities

**Figure 1:**
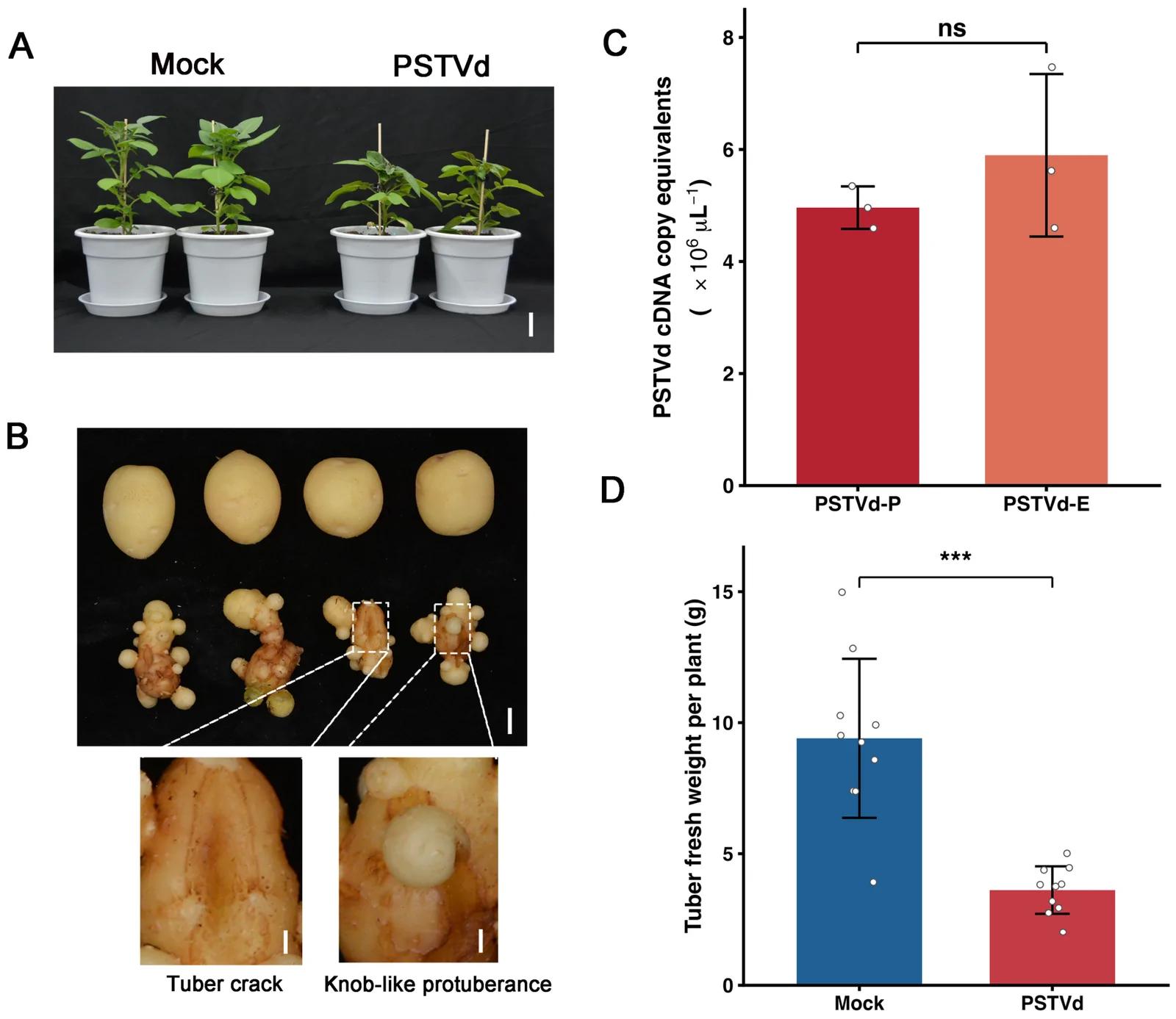
Effects of PSTVd infection on plant and tuber phenotypes of potato K18. (**A**) Plant phenotypes at 21 days after inoculation. Scale bar = 3 cm. (**B**) Tuber phenotypes at harvest and enlarged views of tuber cracks and knob-like protuberances at tuber eyes. Scale bars: upper panel = 1 cm; enlarged images = 2 mm. (**C**) PSTVd cDNA copy equivalents in PSTVd–P and PSTVd–E tissues estimated by probe-based qPCR using a recombinant plasmid DNA standard curve after reverse transcription of equal total-RNA inputs. (**D**) Fresh tuber weight per plant. PSTVd-P and PSTVd-E denote crack regions and regions of knob-like protuberances at tuber eyes of PSTVd-infected tubers, respectively. Data are presented as the mean ± SD; dots represent biological replicates. For (**C**), n = 3 biological replicates per group, with each replicate consisting of region-specific tuber tissues pooled in equal amounts from three independent PSTVd-infected plants; *p* = 0.469, paired two-sided *t*-test; for (**D**), n = 10 plants per group. *** *p* < 0.001; ns, not significant.

At 21 days after PSTVd inoculation, growth of K18 plants was markedly inhibited, as indicated by bunching of the apical leaves, reduced plant height, and a compact plant architecture (Figure 1A). At harvest, mock-inoculated tubers had smooth surfaces and relatively regular shapes, whereas PSTVd-infected tubers were substantially smaller, elongated or spindle-shaped, and displayed pronounced tuber cracks and knob-like protuberances at tuber eyes (Figure 1B). All 30 PSTVd-inoculated plants exhibited both abnormalities (30/30, 100%), whereas neither abnormality was observed in any of the 30 mock-inoculated plants (0/30, 0%).

Probe-based RT-qPCR was used to estimate PSTVd cDNA copy equivalents in both PSTVd-P and PSTVd-E tissues. Using equal total-RNA inputs, the estimated PSTVd cDNA copy equivalents did not differ significantly between PSTVd-P and PSTVd-E (*p* = 0.469, paired two-sided *t*-test; Figure 1C). PSTVd infection also significantly reduced fresh tuber weight per plant (Figure 1D), demonstrating that infection altered tuber morphology while strongly suppressing tuber yield per plant.

### 2.2. RNA Sequencing Reveals Region-Dependent Transcriptomic States in PSTVd-Infected Tubers

To characterize the transcriptional states of different phenotypic regions in PSTVd-infected potato tubers, RNA sequencing was performed on four tissue groups: Mock-P (smooth surface regions of mock-inoculated tubers), Mock-E (normal eye tissues of mock-inoculated tubers), PSTVd-P (crack regions of PSTVd-infected tubers), and PSTVd-E (regions of knob-like protuberances at tuber eyes of PSTVd-infected tubers). Quality assessment showed that clean data accounted for more than 98.67% of the reads in every sample. Q20 and Q30 base percentages were at least 97.26% and 92.36%, respectively, and GC content ranged from 41.78% to 42.78%, indicating high sequencing quality (Table S1). Correlation analysis showed high overall similarity among biological replicates within each group, supporting the reproducibility of the RNA-seq data and their suitability for downstream analyses (Figure S1).

**Figure 2:**
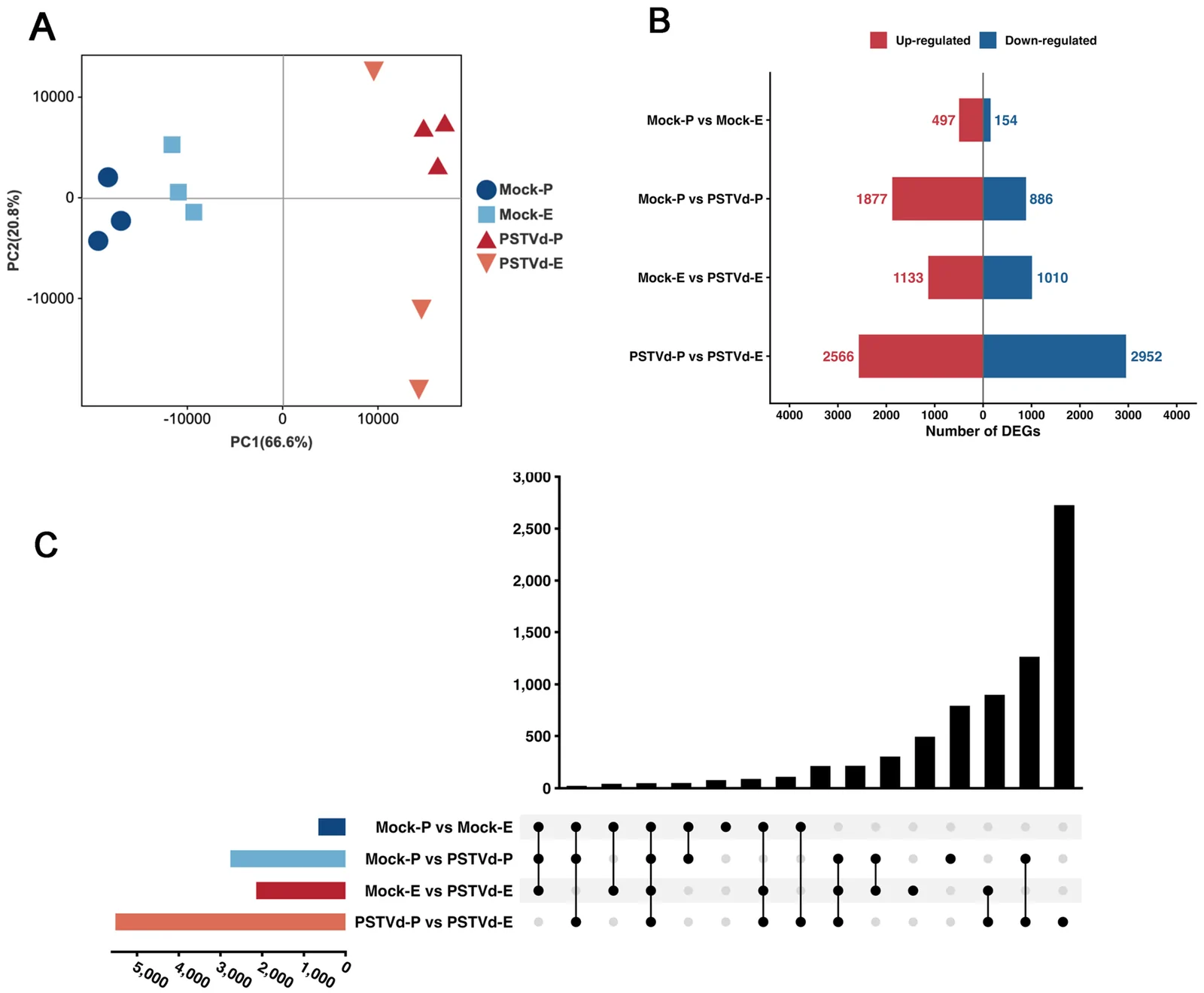
PSTVd-induced local transcriptomic reprogramming in potato tubers. (**A**) Principal component analysis of global gene-expression profiles in the four sample groups. (**B**) Numbers of upregulated and downregulated differentially expressed genes (DEGs) in the four comparisons; the direction of change indicates the second group relative to the first. (**C**) UpSet analysis of shared and comparison-specific DEGs. Mock-P and Mock-E denote smooth surface regions and normal eye tissues of mock-inoculated tubers, respectively; PSTVd-P and PSTVd-E denote crack regions and regions of knob-like protuberances at tuber eyes of PSTVd-infected tubers, respectively.

Principal component analysis (PCA) of the global gene-expression profiles showed that PC1 and PC2 explained 66.6% and 20.8% of the total variance, respectively (Figure 2A). Mock-P and Mock-E samples were located primarily on the negative side of PC1, whereas PSTVd-P and PSTVd-E samples were located primarily on the positive side, indicating that PSTVd infection was associated with major transcriptomic separation among the samples. Mock-P and Mock-E also showed partial separation, reflecting distinct basal transcriptional features of smooth surface regions and normal eye tissues in mock-inoculated tubers. After infection, PSTVd-P and PSTVd-E tended to separate in the PC1-PC2 plane, suggesting differences in the local transcriptional profiles of crack regions and regions of knob-like protuberances at tuber eyes.

Using FDR < 0.05 and |log_2_(fold change)| > 1 as thresholds, 651, 2763, 2143, and 5518 differentially expressed genes (DEGs) were identified in the Mock-P vs. Mock-E, Mock-P vs. PSTVd-P, Mock-E vs. PSTVd-E, and PSTVd-P vs. PSTVd-E comparisons, respectively (Figure 2B; Table S2). The corresponding numbers of upregulated and downregulated DEGs were 497 and 154, 1877 and 886, 1133 and 1010, and 2566 and 2952, respectively. Throughout this manuscript, upregulation and downregulation refer to expression in the second group relative to the first group in each comparison. The PSTVd-P vs. PSTVd-E comparison yielded the largest number of DEGs, indicating extensive transcriptional differences between the two abnormal tuber regions.

UpSet analysis identified both shared DEGs and numerous comparison-specific DEGs across the four comparisons (Figure 2C). The PSTVd-P vs. PSTVd-E comparison contained the largest number of comparison-specific DEGs, further demonstrating distinct transcriptional regulation in crack regions and regions of knob-like protuberances at tuber eyes. Collectively, PSTVd infection was associated with distinct transcriptomic states in local tuber tissues and different gene-expression patterns between the two abnormal regions, providing a basis for subsequent analysis of their molecular features.

### 2.3. Defense Responses and Cell Wall Remodeling Associated with Tuber Crack Regions After PSTVd Infection

**Figure 3:**
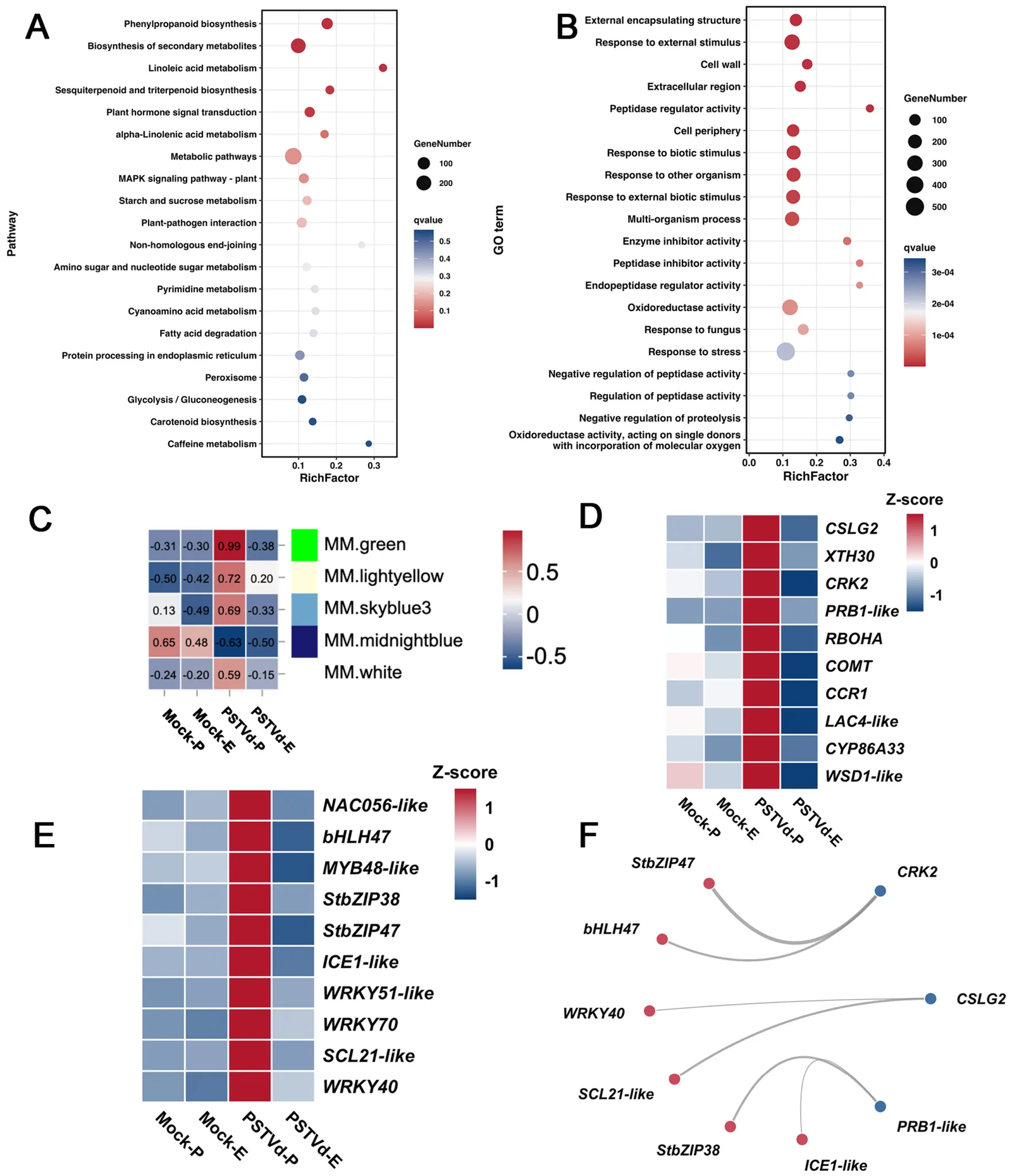
Defense responses, cell wall remodeling, and candidate co-expression networks in PSTVd-infected potato tuber crack regions. (**A**,**B**) KEGG (**A**) and GO (**B**) enrichment analyses of DEGs in the Mock-P vs. PSTVd-P comparison. Bubble size indicates the number of enriched genes, color indicates the q value, and Rich Factor indicates the proportion of enriched genes. (**C**) Heatmap of correlations between WGCNA modules and sample types. Values represent Pearson correlation coefficients. (**D**,**E**) Expression heatmaps of representative candidate genes (**D**) and transcription factors (**E**) in the green module; colors represent row Z-scores. (**F**) Co-expression network of candidate transcription factors and representative candidate genes. Red and blue nodes represent transcription factors and representative candidate genes, respectively, and edge width indicates association strength. Mock-P and Mock-E denote smooth surface regions and normal eye tissues of mock-inoculated tubers, respectively; PSTVd-P and PSTVd-E denote crack regions and regions of knob-like protuberances at tuber eyes of PSTVd-infected tubers, respectively.

To characterize molecular responses associated with PSTVd-induced tuber cracking, KEGG and GO enrichment analyses were first performed on DEGs from the Mock-P vs. PSTVd-P comparison; complete results are provided in Table S3. KEGG analysis showed significant enrichment of phenylpropanoid biosynthesis, biosynthesis of secondary metabolites, linoleic acid metabolism, sesquiterpenoid and triterpenoid biosynthesis, and plant hormone signal transduction (q < 0.05) (Figure 3A). Alpha-linolenic acid metabolism, MAPK signaling pathway, starch and sucrose metabolism, and plant-pathogen interaction also ranked highly but did not remain significant after multiple-testing correction. GO analysis further showed significant enrichment of external encapsulating structure, cell wall, extracellular region, cell periphery, response to external stimulus, response to biotic stimulus, response to stress, and oxidoreductase activity (Figure 3B). Thus, distinct transcriptional features were observed in crack regions after PSTVd infection, involving the cell wall and extracellular structures, phenylpropanoid and secondary metabolism, linoleic acid and terpenoid metabolism, hormone signaling, responses to external and biotic stimuli, and oxidoreductase activity.

To identify co-expression modules associated with tuber cracking, WGCNA was performed using the RNA-seq expression matrix. Based on changes in the scale-free topology fit index and mean connectivity, β = 14 was selected as the soft-thresholding power for network construction (Figure S2A). Gene clustering and module assignment are shown in Figure S2B, and the complete module-trait correlations are provided in Figure S2C and Table S4. The green module showed the strongest positive correlation with PSTVd-P (r = 0.994) (Figure 3C; Figure S2C) and was therefore designated as the candidate PSTVd-P associated module for subsequent selection of candidate genes and associated transcription factors.

Representative candidate genes were selected from the intersection between green-module genes and DEGs in crack regions on the basis of intramodular connectivity, functional annotation, and phenotype relevance. These genes included *CSLG2*, *XTH30*, *CRK2*, *PRB1-like*, *RBOHA*, *COMT*, *CCR1*, *LAC4-like*, *CYP86A33*, and *WSD1-like* (Figure 3D; Table S4), and they generally showed higher relative expression in PSTVd-P. *CSLG2* and *XTH30* were annotated as a cellulose synthase-like protein and a xyloglucan endotransglucosylase/hydrolase protein, respectively, suggesting potential associations with cell wall polysaccharide synthesis and remodeling. *CRK2*, *PRB1-like*, and *RBOHA* are primarily associated with receptor-kinase signaling, defense, and ROS responses; *COMT*, *CCR1*, and *LAC4-like* are associated with phenylpropanoid and lignin metabolism; and *CYP86A33* and *WSD1-like* are related to fatty acid hydroxylation and surface barrier metabolism, including wax formation.

Candidate transcription factors identified in the green module included *NAC056-like*, *bHLH47*, *MYB48-like*, *StbZIP38*, *StbZIP47*, *ICE1-like*, *WRKY51-like*, *WRKY70*, *SCL21-like*, and *WRKY40* (Figure 3E; Table S4). A TF-candidate gene co-expression network constructed from co-expression relationships showed associations of *StbZIP47* and *bHLH47* with *CRK2*, *SCL21-like* and *WRKY40* with *CSLG2*, and *StbZIP38* and *ICE1-like* with *PRB1-like* (Figure 3F; Table S4). These relationships indicate coordinated expression between the candidate transcription factors and genes involved in defense responses and cell wall remodeling in crack regions, suggesting potential associations rather than confirmed regulatory relationships. In summary, the transcriptional features observed in crack regions after PSTVd infection involved cell wall and surface barrier related metabolism, phenylpropanoid and lipid metabolism, hormone signaling, defense, and redox responses, together with a trend toward enrichment of the MAPK signaling pathway. WGCNA and co-expression analysis further supported the green module as a candidate PSTVd-P associated module and suggested that *CRK2*, *CSLG2*, and *PRB1-like* were co-expressed with WRKY, bHLH, GRAS, and bZIP transcription factors in crack regions after PSTVd infection. The associations of other module-associated transcription factors, including *NAC056-like*, require further validation.

### 2.4. Development and Growth-Related Transcriptional Features Associated with Knob-like Protuberances at Tuber Eyes After PSTVd Infection

**Figure 4:**
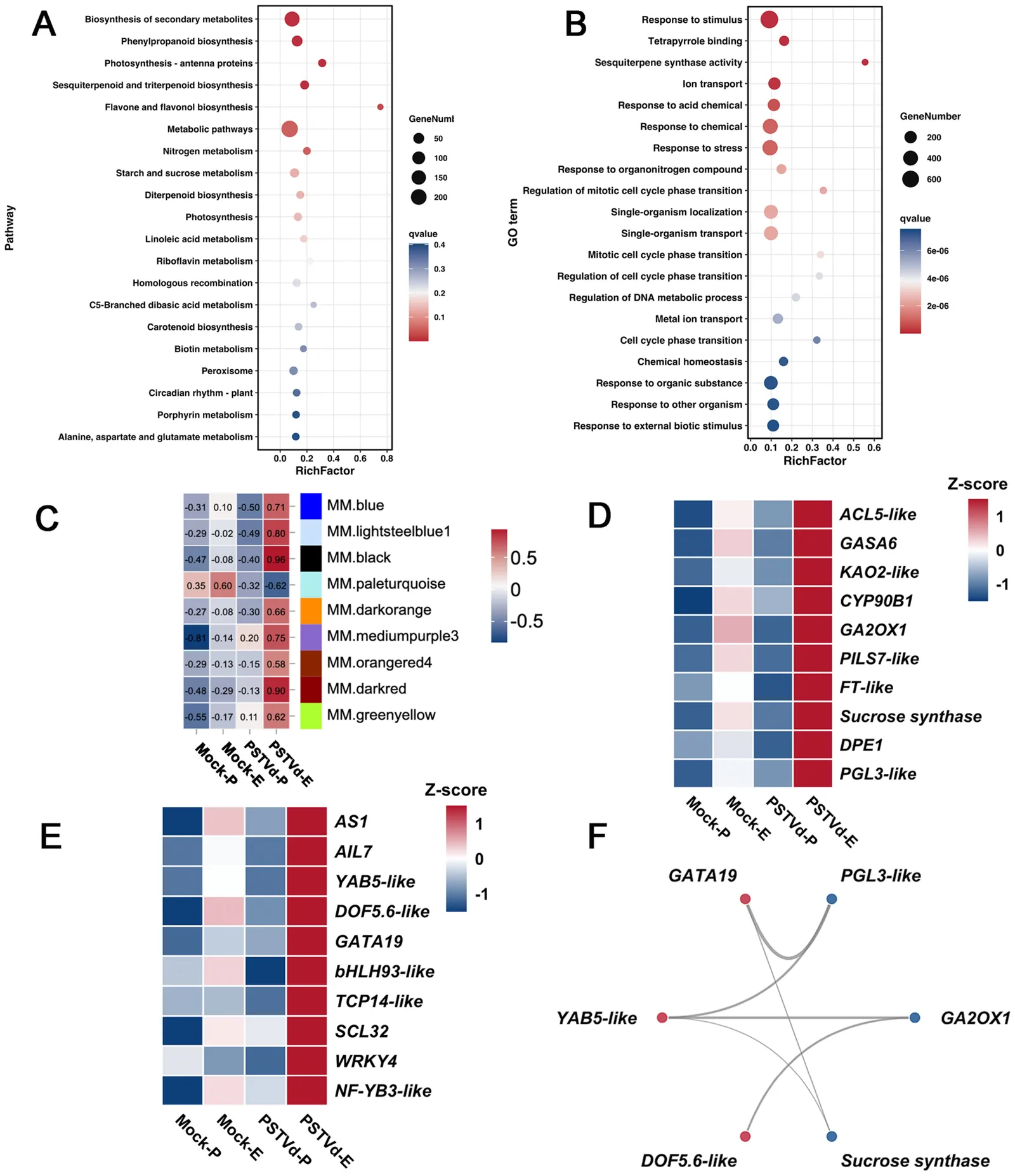
Transcriptional reprogramming and candidate co-expression networks in regions of knob-like protuberances at potato tuber eyes after PSTVd infection. (**A**,**B**) KEGG (**A**) and GO (**B**) enrichment analyses of DEGs in the Mock-E vs. PSTVd-E comparison. Bubble size indicates the number of enriched genes, color indicates the q value, and Rich Factor indicates the enrichment proportion. (**C**) Heatmap of correlations between WGCNA modules and sample types. Values represent Pearson correlation coefficients. (**D**,**E**) Expression heatmaps of representative candidate genes (**D**) and transcription factors (**E**) in the black module; colors represent row Z-scores. (**F**) Co-expression network of candidate transcription factors and representative candidate genes. Red and blue nodes represent transcription factors and representative candidate genes, respectively, and edge width indicates association strength. Mock-P and Mock-E denote smooth surface regions and normal eye tissues of mock-inoculated tubers, respectively; PSTVd-P and PSTVd-E denote crack regions and regions of knob-like protuberances at tuber eyes of PSTVd-infected tubers, respectively.

To characterize molecular responses associated with knob-like protuberances at tuber eyes after PSTVd infection, KEGG and GO enrichment analyses were performed on DEGs from the Mock-E vs. PSTVd-E comparison; complete results are provided in Table S3. The highest-ranking KEGG pathways included biosynthesis of secondary metabolites, phenylpropanoid biosynthesis, photosynthesis-antenna proteins, sesquiterpenoid and triterpenoid biosynthesis, flavone and flavonol biosynthesis, nitrogen metabolism, starch and sucrose metabolism, diterpenoid biosynthesis, and photosynthesis (Figure 4A). These results indicate broad transcriptional changes in secondary metabolism, carbon and nitrogen metabolism, photosynthesis-related processes, and terpenoid metabolism in regions of knob-like protuberances at tuber eyes.

GO enrichment analysis showed that DEGs were primarily enriched in response to stimulus, response to chemical, response to stress, ion transport, single-organism localization, single-organism transport, regulation of mitotic cell cycle phase transition, mitotic cell cycle phase transition, regulation of DNA metabolic process, cell cycle phase transition, and chemical homeostasis (Figure 4B). In contrast to the predominant cell wall/extracellular structure changes and defense responses in crack regions, regions of knob-like protuberances at tuber eyes showed more pronounced changes in the cell cycle, DNA metabolism, transport, and chemical homeostasis, suggesting altered expression patterns related to cell cycle activity and developmental states in local eye tissues.

To identify co-expression modules associated with knob-like protuberances at tuber eyes, WGCNA module-trait correlation analysis was performed. Complete network construction and module-trait correlation results are shown in Figure S2 and Table S5. The black, darkred, lightsteelblue1, mediumpurple3, blue, darkorange, and greenyellow modules were positively correlated with PSTVd-E, with the black module showing the strongest association (r = 0.96) (Figure 4C; Figure S2C). The black module was therefore selected as the candidate module associated with knob-like protuberances at tuber eyes for subsequent identification of candidate genes and associated transcription factors.

Representative candidate genes were selected from the intersection between black-module genes and DEGs in regions of knob-like protuberances at tuber eyes on the basis of intramodular connectivity, functional annotation, and phenotype relevance. These genes included *ACL5-like*, *GASA6*, *KAO2-like*, *CYP90B1*, *GA2OX1*, *PILS7-like*, *FT-like*, *Sucrose synthase*, *DPE1*, and *PGL3-like* and generally showed higher expression in PSTVd-E (Figure 4D; Table S5). *ACL5-like* is associated with thermospermine/polyamine metabolism, cell elongation, and developmental regulation; *GASA6* is a GA-responsive protein; *KAO2-like* and *GA2OX1* may participate in GA biosynthesis and inactivation, respectively; *CYP90B1* is associated with brassinosteroid biosynthesis; and *PILS7-like* is associated with intracellular auxin transport and homeostasis. In addition, *FT-like* is associated with developmental transitions, *Sucrose synthase* and *DPE1* are associated with carbohydrate metabolism, and *PGL3-like* may contribute to cell wall remodeling. These findings indicate coordinated transcriptional changes associated with hormone-related growth regulation, carbon metabolism, and cell wall processes in regions of knob-like protuberances at tuber eyes.

Candidate transcription factors identified in the black module included *AS1*, *AIL7*, *YAB5-like*, *DOF5.6-like*, *GATA19*, *bHLH93-like*, *TCP14-like*, *SCL32*, *WRKY4*, and *NF-YB3-like* (Figure 4E; Table S5). These candidates generally showed higher expression in PSTVd-E and represented transcription factor families associated with organ development, cell cycle regulation, hormone responses, and stress regulation. A transcription factor candidate gene co-expression network showed co-expression associations of *YAB5-like*, *DOF5.6-like*, and *GATA19* with representative candidate genes such as *GA2OX1*, *PGL3-like*, and *Sucrose synthase* (Figure 4F; Table S5). These associations suggest that the corresponding transcription factors show co-expression relationships with genes involved in GA metabolism, carbohydrate metabolism, and cell wall remodeling in regions of knob-like protuberances at tuber eyes, although direct regulatory relationships require experimental validation.

In summary, regions of knob-like protuberances at tuber eyes showed transcriptional responses distinct from those in crack regions after PSTVd infection. The major changes involved the cell cycle and DNA metabolism, transport, and carbon metabolism and were accompanied by altered expression of candidate genes associated with GA, brassinosteroids, and auxin. WGCNA and co-expression analysis further supported the black module as a candidate PSTVd-E-associated module and identified *ACL5-like*, *GA2OX1*, *PILS7-like*, *GASA6*, *Sucrose synthase*, and *PGL3-like*, together with the candidate transcription factors *YAB5-like*, *DOF5.6-like*, and *GATA19*, as genes associated with transcriptional changes related to growth and developmental processes in the eye region. Their direct regulatory relationships and functions in protuberance formation remain to be established.

### 2.5. Infection × Region Interaction Analysis Reveals Distinct Transcriptional Responses Associated with Tuber Cracking and Knob-like Protuberances at Tuber Eyes

**Figure 5:**
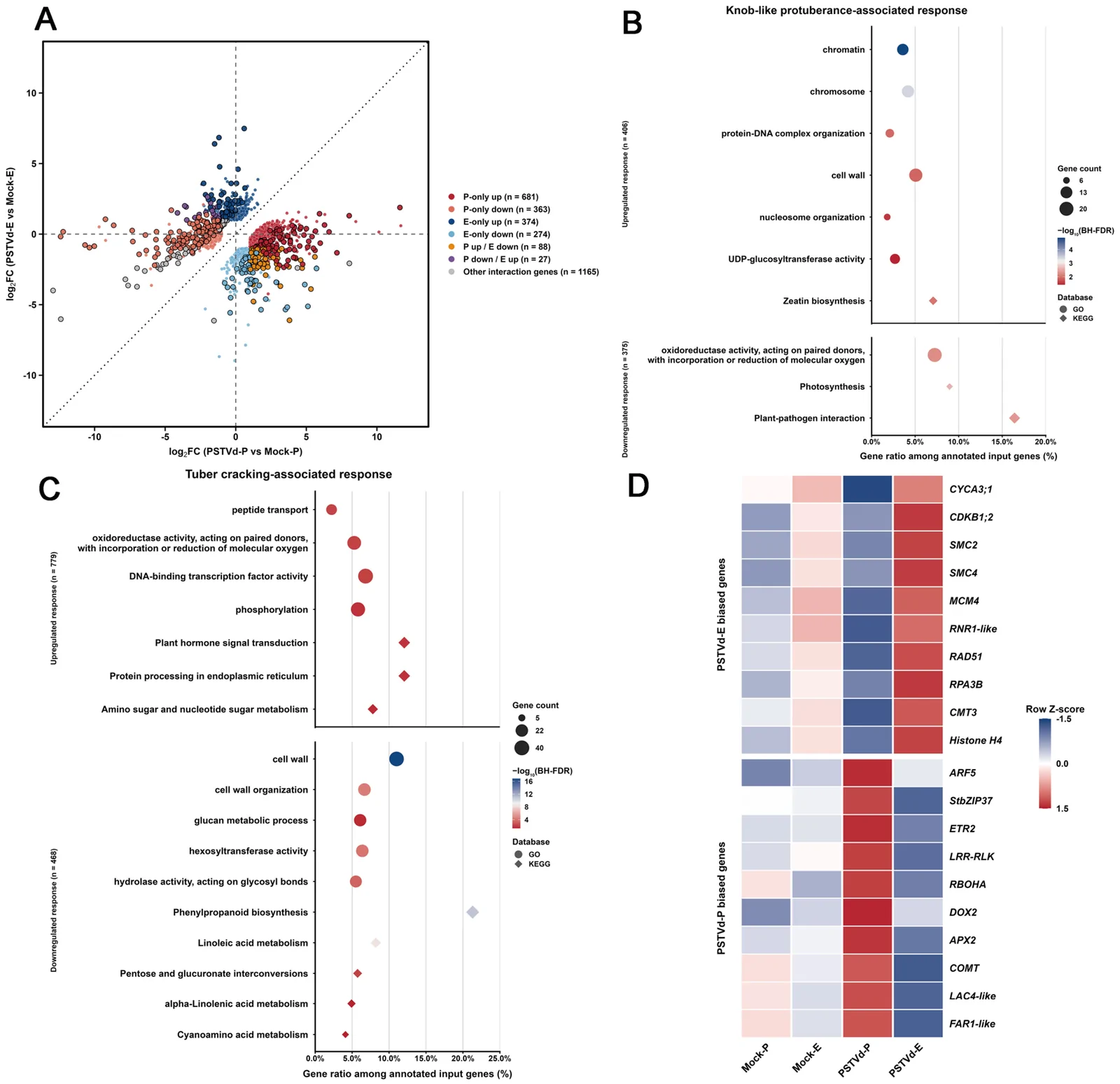
Region-dependent transcriptional responses induced by PSTVd infection. (**A**) Distribution of response types among genes with infection × region interaction effects. (**B**,**C**) GO and KEGG enrichment analyses of directional gene sets associated with knob-like protuberances at tuber eyes (**B**) and tuber cracking (**C**). Circles and diamonds represent GO and KEGG terms, respectively; point size indicates gene number, and color indicates −log_10_(BH-FDR). (**D**) Expression heatmap of representative PSTVd-E and PSTVd-P biased response genes; colors represent row Z-scores.

To distinguish the transcriptional responses to PSTVd infection in different tuber regions, an interaction analysis between infection and sampling region was performed. A two-factor model was fitted to the Mock-P, Mock-E, PSTVd-P, and PSTVd-E samples, identifying 2972 genes with significant infection × region interaction effects (FDR < 0.05 and |interaction log_2_(fold change)| ≥ 1; Figure 5A; Table S6). Among these genes, 681 and 363 were upregulated and downregulated only in the P region, respectively, whereas 374 and 274 were upregulated and downregulated only in the E region. An additional 88 genes were upregulated in the P region and downregulated in the E region, 27 showed the opposite pattern, and the remaining 1165 displayed other forms of region-dependent response. These results demonstrate that the effects of PSTVd infection on the tuber transcriptome are strongly region-dependent.

Enrichment analysis of directionally classified genes associated with knob-like protuberances at tuber eyes showed that upregulated responses were primarily related to the organization of chromatin, chromosomes, and protein-DNA complexes, nucleosome organization, the cell wall, UDP-glycosyltransferase activity, and zeatin biosynthesis, whereas downregulated responses were mainly related to oxidoreductase activity, photosynthesis, and plant-pathogen interaction pathways (Figure 5B). In contrast, upregulated responses associated with tuber cracking were enriched in peptide transport, oxidoreductase activity, DNA-binding transcription factor activity, protein phosphorylation, plant hormone signal transduction, protein processing in the endoplasmic reticulum, and amino-sugar and nucleotide-sugar metabolism. Downregulated responses were significantly associated with the cell wall and cell wall organization, glucan metabolism, glycosyl transfer and hydrolysis of glycosidic bonds, phenylpropanoid biosynthesis, linoleic and alpha-linolenic acid metabolism, and pentose and glucuronate interconversions (Figure 5C). These results indicate that the two symptom regions displayed distinct transcriptional programs characterized, respectively, by processes related to the cell cycle, DNA replication, and chromatin organization, and by responses related to the cell wall, lipid metabolism, and defense.

Pre-ranked gene set enrichment analysis (GSEA) further supported these coordinated region-biased changes. Positive enrichment represented PSTVd-E biased responses, with significant enrichment of mitotic cell cycle phase transition, DNA replication, nucleosome organization, and the KEGG DNA replication pathway. Negative enrichment represented PSTVd-P biased responses and included plant hormone signal transduction, the plant MAPK signaling pathway, response to oxidative stress, and flavonoid metabolic processes (Figure S3). The normalized enrichment scores for cell cycle phase transition and nucleosome organization were 2.01 and 2.08, respectively, indicating that genes related to cell cycle regulation and chromatin reorganization showed a coordinated E-region bias across the ranked gene list rather than being driven by a small number of DEGs.

To visualize the expression patterns of genes with interaction effects, 20 representative region-biased genes were selected for clustered heatmap analysis (Figure 5D). *CYCA3;1*, *CDKB1;2*, *SMC2*, *SMC4*, *MCM4*, *RNR1-like*, *RAD51*, *RPA3B*, *CMT3*, and *Histone H4* generally showed PSTVd-E biased responses and were associated with the cell cycle, DNA replication, chromosome maintenance, and chromatin organization. *ARF5*, *StbZIP37*, *ETR2*, *LRR-RLK*, *RBOHA*, *DOX2*, *APX2*, *COMT*, *LAC4-like*, and *FAR1-like* generally showed PSTVd-P biased responses and were associated with hormone and defense signaling, ROS homeostasis, phenylpropanoid metabolism, cell wall construction, and lipid barrier formation. Here, ‘PSTVd-E biased’ and ‘PSTVd-P biased’ refer to the direction of the infection × region interaction and do not imply that every gene was simply upregulated in the corresponding region. Overall, interaction analysis revealed clearly distinct yet internally coordinated transcriptional reprogramming in tuber crack regions and regions of knob-like protuberances at tuber eyes.

### 2.6. Crack Regions and Regions of Knob-like Protuberances at Tuber Eyes Display Distinct Phytohormone Metabolic Profiles After PSTVd Infection

**Figure 6:**
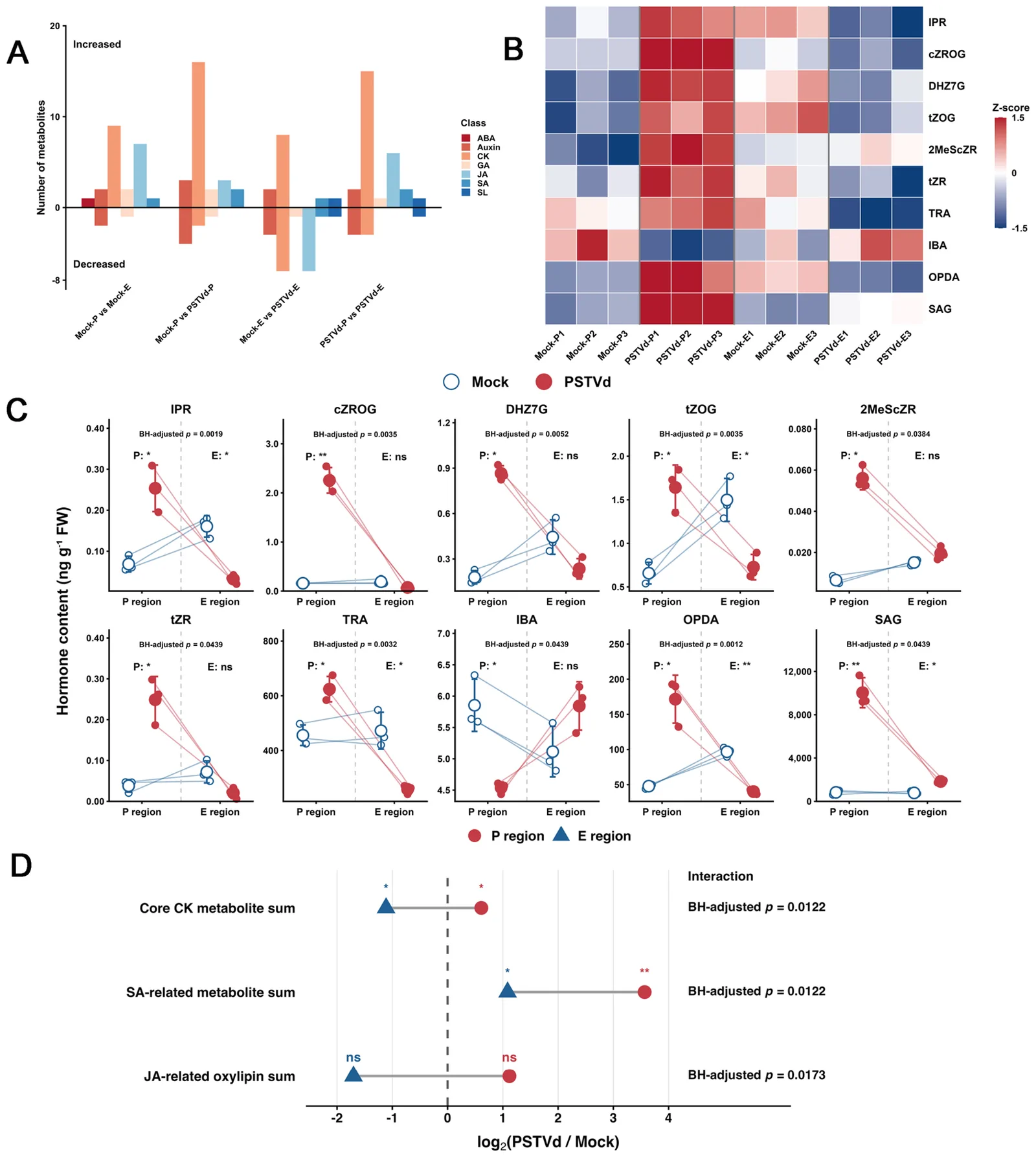
Region-dependent changes in phytohormone metabolism in potato tubers after PSTVd infection. (**A**) Number of metabolites exceeding the predefined fold-change threshold (fold change ≥ 2 or ≤0.5), classified as increased or decreased according to the direction of change, in each hormone class across the comparisons. (**B**) Heatmap of 10 metabolites with significant infection × region interaction effects; data were log_2_-transformed and standardized as row Z-scores. (**C**) Contents of the 10 metabolites in the P and E regions. Small points represent biological replicates, large points represent means, and error bars represent SD. Lines connect paired P- and E-region samples derived from the same biological plant pool. (**D**) log_2_(PSTVd/Mock) values for the molar sums of core CK metabolites, SA-related metabolites, and JA-related oxylipins. Symbols following P and E indicate the simple effects of PSTVd versus Mock within the corresponding region. BH-adjusted *p* values shown in panels C and D correspond to the infection × region interaction. *p* values for the interaction and the P- and E-region simple effect test families were adjusted separately using the Benjamini–Hochberg procedure. n = 3; * BH-adjusted *p* < 0.05, ** BH-adjusted *p* < 0.01, ns, BH-adjusted *p* ≥ 0.05.

To determine whether changes in hormone metabolism are associated with PSTVd-induced tuber cracking and knob-like protuberances at tuber eyes, phytohormone metabolomic analysis was performed on Mock-P, Mock-E, PSTVd-P, and PSTVd-E samples. Metabolites exceeding the predefined fold-change threshold (fold change ≥ 2 or ≤0.5) were identified in each comparison and classified as increased or decreased according to the direction of change, with distinct patterns among hormone classes (Figure 6A). Venn analysis further revealed both shared and comparison-specific metabolites exceeding this threshold (Figure S4B). Exploratory KEGG enrichment analysis of these metabolites indicated associations with plant hormone signal transduction, zeatin biosynthesis, tryptophan metabolism, alpha-linolenic acid metabolism, and carotenoid biosynthesis (Figure S4C–F). These results show that PSTVd infection not only altered the overall hormone-metabolic state of tubers but also produced region-dependent effects.

To identify region-dependent hormone changes associated with the two localized symptoms, infection × region interactions were evaluated using paired regional contrasts between the P and E regions, as described in the Methods. Ten hormone-related metabolites showed significant infection × region interaction effects after Benjamini–Hochberg correction (BH-adjusted *p* < 0.05): IPR, cZROG, DHZ7G, tZOG, 2MeScZR, tZR, TRA, IBA, OPDA, and SAG (Figure 6B,C). These metabolites displayed clear region-dependent accumulation patterns across the four groups.

Several CK-related metabolites, including IPR, cZROG, DHZ7G, tZOG, 2MeScZR, and tZR, generally increased in the P region after PSTVd infection but markedly decreased in the E region. TRA and OPDA showed similar patterns, accumulating in the P region and decreasing in the E region. In contrast, IBA decreased in the P region and increased in the E region. SAG increased after infection in both regions, but the increase was substantially greater in the P region. Thus, the effect of PSTVd on tuber hormone metabolism was not a uniform overall increase or decrease; instead, distinct metabolic patterns were associated with crack regions and regions of knob-like protuberances at tuber eyes.

Because individual metabolites reflect only part of a hormone-metabolic pathway, relevant compounds were converted to molar contents, and the molar sums of core CK metabolites, SA-related metabolites, and JA-related oxylipins were calculated. The molar sum of core CK metabolites showed a significant infection × region interaction (BH-adjusted *p* = 0.0122), increasing significantly after PSTVd infection in the P region and decreasing significantly in the E region (Figure 6D). The molar sum of SA-related metabolites also showed a significant infection × region interaction (BH-adjusted *p* = 0.0122). It increased after infection in both regions, but the magnitude of accumulation was substantially greater in the P region. The molar sum of JA-related oxylipins likewise showed a significant infection × region interaction (BH-adjusted *p* = 0.0173), with an overall tendency to increase in the P region and decrease in the E region, although the simple effect within each region was not significant after BH correction.

Further analysis of major free hormones and their precursors showed that IAA, 1-aminocyclopropane-1-carboxylic acid (ACC), and SA increased significantly after PSTVd infection in the P region but did not change significantly in the E region. In contrast, the simple effects of JA, jasmonoyl-L-isoleucine (JA-Ile), and ABA were not significant in either region. Because most of these individual compounds did not show significant infection × region interactions, their changes primarily reflect local infection responses. Region-specific conclusions are therefore supported mainly by metabolites with significant interaction effects and by the molar-sum results (Figure S5).

Collectively, PSTVd infection broadly reprogrammed tuber hormone metabolism and generated distinct hormonal environments in crack regions and regions of knob-like protuberances at tuber eyes. Crack regions were characterized primarily by accumulation of several CK-related metabolites, SA-related metabolites, and OPDA, whereas regions of knob-like protuberances at tuber eyes showed decreases in several CK-related metabolites and an overall downward trend in JA-related oxylipins. These findings indicate distinct region-dependent hormone-metabolic states associated with the two localized symptomatic regions; however, they do not establish a causal role for these hormone changes in symptom formation.

### 2.7. Integrated Transcriptome-Phytohormone Analysis

**Figure 7:**
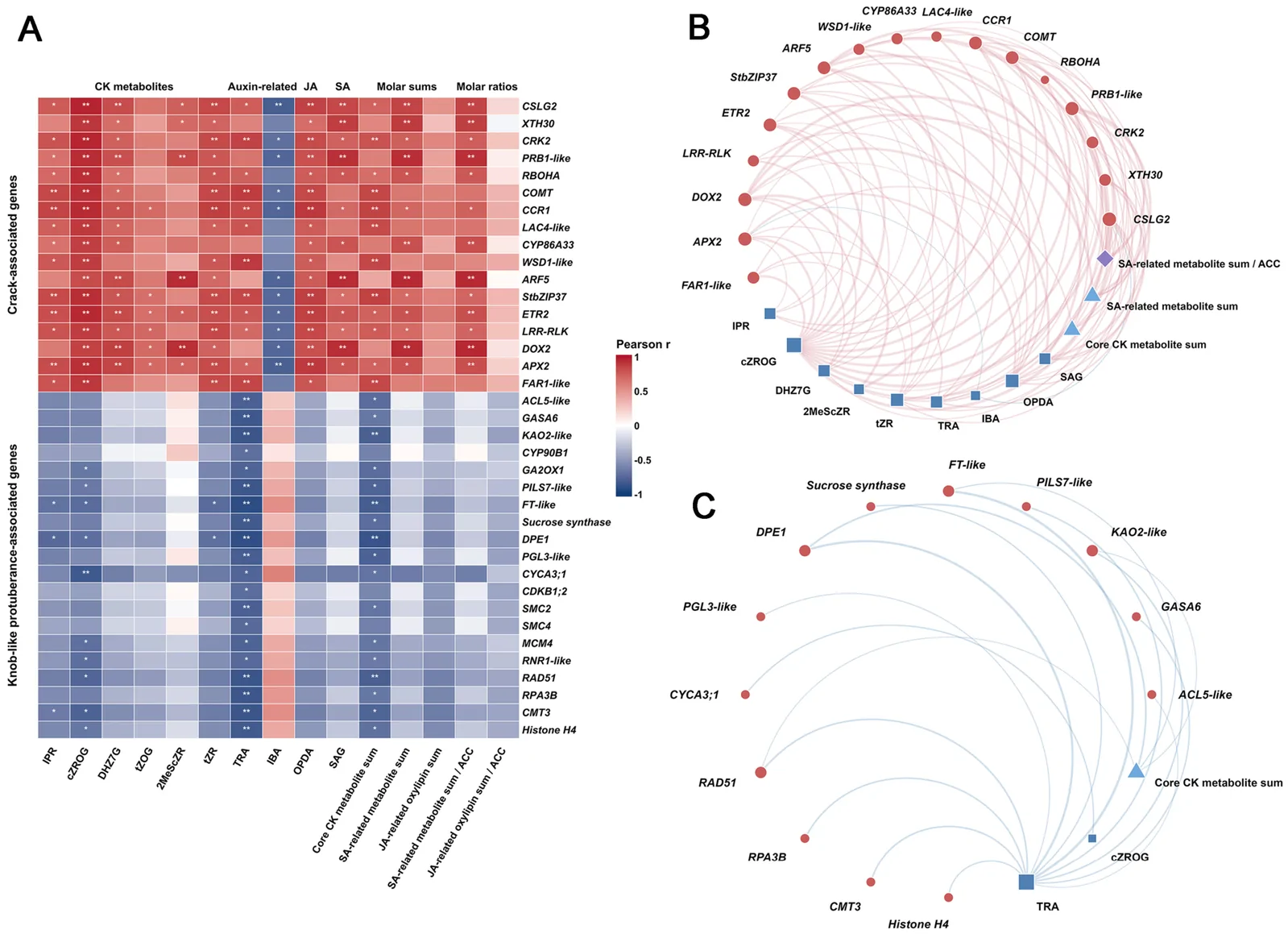
Integrated gene–hormone analysis of tuber cracking and knob-like protuberances at tuber eyes. (**A**) Pearson correlation heatmap of representative phenotype-related genes with key hormone metabolites, molar sums, and molar ratios. Red and blue indicate positive and negative Pearson correlation coefficients, respectively. *p* values from all 37 × 15 = 555 gene–hormone tests were adjusted together using the Benjamini–Hochberg method; * and ** indicate q < 0.05 and q < 0.01, respectively. (**B**,**C**) Gene-hormone association networks for tuber crack regions (**B**) and regions of knob-like protuberances at tuber eyes (**C**), showing only associations with |r| ≥ 0.80 and BH-FDR-adjusted q < 0.05. Different shapes represent candidate genes, hormone metabolites, molar sums, and molar ratios. Red and blue edges indicate positive and negative correlations, respectively, and edge width indicates correlation strength. Because the analysis was based on only 12 matched biological samples, these gene-hormone networks should be regarded as exploratory.

To examine exploratory associations between hormone changes and the expression of phenotype-related genes after PSTVd infection, Pearson correlation analysis was performed between 37 candidate genes and 15 hormone-related indices, yielding 555 gene–hormone tests (Table S8). *p* values from all 555 tests were globally adjusted using the Benjamini–Hochberg method. The correlation heatmap showed that genes associated with tuber cracking and PSTVd-P biased responses were generally positively correlated with multiple CK metabolites, SA-related indices, and selected molar sums and molar ratios, whereas genes associated with knob-like protuberances at tuber eyes and PSTVd-E biased responses generally showed the opposite pattern (Figure 7A). Genes related to cell wall remodeling, phenylpropanoid metabolism, ROS-related responses, and defense in crack regions tended to show positive associations with several hormone-related indices, whereas genes related to hormone metabolism, carbohydrate metabolism, cell cycle regulation, and chromatin organization in protuberance regions tended to show negative associations. These results indicate distinct, but exploratory, transcript-hormone association patterns in the two abnormal tuber regions.

Gene-hormone association networks were then constructed using thresholds of |r| ≥ 0.80 and BH-FDR-adjusted q < 0.05. Crack-associated genes formed a relatively dense network with CK-related metabolites, IBA, OPDA, SAG, and SA-related indices (Figure 7B). *CSLG2*, *XTH30*, *CRK2*, *PRB1-like*, *RBOHA*, *COMT*, *CCR1*, *LAC4-like*, *CYP86A33*, and *WSD1-like* showed associations with hormone-related indices, showing coordinated transcript-hormone associations involving cell wall remodeling, defense-related responses, and hormone-metabolic changes in crack regions. In contrast, the network associated with knob-like protuberances at tuber eyes was substantially sparser, with all 17 retained associations being negative and concentrated on TRA, the core CK metabolite sum, and cZROG (Figure 7C). These associations involved *ACL5-like*, *GASA6*, *KAO2-like*, *PILS7-like*, *FT-like*, *Sucrose synthase*, *DPE1*, *PGL3-like*, and PSTVd-E biased genes including *CYCA3;1*, *RAD51*, *RPA3B*, *CMT3*, and *Histone H4*. This pattern is consistent with the decrease in multiple CK-related metabolites in the E region shown in Figure 6 and indicates that transcriptomic states related to cell cycle and chromatin processes coincided with a distinct CK-related metabolic environment. Because these correlations were calculated from only 12 samples, they should be interpreted as exploratory associations rather than evidence of direct regulatory relationships.

To assess the concordance between RT-qPCR and RNA-seq expression patterns, six genes were examined, including representative candidates highlighted in the preceding analyses (*CSLG2* and *RBOHA* for the crack region, and *ACL5-like* and *PILS7-like* for the knob-like protuberance region) and two additional genes showing region-associated expression patterns (*WRKY6* and *CHTF8-like*). *CSLG2*, *WRKY6*, and *RBOHA* were expressed predominantly in PSTVd-P, whereas *ACL5-like*, *PILS7-like*, and *CHTF8-like* were expressed predominantly in PSTVd-E, broadly consistent with the region-biased patterns obtained by RNA sequencing (Figure S6). Thus, RT-qPCR supported the region-biased expression patterns of these selected genes.

## 3. Discussion

This study showed that PSTVd infection markedly inhibited growth and tuber development in potato K18 and induced tuber cracking and knob-like protuberances at tuber eyes, consistent with typical PSTVd symptoms reported previously [6,8,9]. PSTVd cDNA copy equivalent estimates did not differ significantly between PSTVd-P and PSTVd-E under the present assay conditions. Because these estimates were derived using a plasmid DNA standard curve after reverse transcription of equal amounts of total RNA and were not normalized to cell number or a stable host marker, they should not be interpreted as absolute PSTVd RNA copy numbers or cellular viroid loads. The two symptomatic regions differ morphologically and may also differ in cell composition and density; therefore, the absence of a significant difference indicates only similar PSTVd cDNA copy equivalent estimates under the present equal RNA input assay. Moreover, these measurements were obtained only at the 80-dpi sampling time; therefore, the absence of a significant difference between PSTVd-P and PSTVd-E cannot exclude earlier region-specific differences in PSTVd accumulation before visible symptom development. Similar PSTVd cDNA copy-equivalent estimates in the two symptomatic regions do not necessarily imply identical viroid sequence composition. Previous studies have shown that specific nucleotide substitutions, particularly within pathogenicity-associated regions of PSTVd, can alter pathogenicity and symptom expression [33,34,35]. To further examine this possibility, full-length PSTVd amplicons from three biological replicates of each symptomatic region were subjected to bidirectional Sanger sequencing. No nucleotide differences were detected at the consensus-sequence level between the PSTVd-P and PSTVd-E samples, and the consensus sequences of all six samples were concordant with the inoculated PSTVd-S sequence (GenBank accession X58388.1; Supplementary File S1). However, because direct Sanger sequencing primarily reflects the predominant sequence population, low-frequency PSTVd sequence variants cannot be excluded. The distinct molecular states observed in the two regions may reflect both region-dependent responses to PSTVd infection and inherent differences in the developmental, morphological, and physiological states of the sampled tissues. Although the infection × region interaction analysis helps identify region-dependent infection effects statistically, the present experimental design cannot fully disentangle infection-related effects from differences inherent to the tissue types being compared. PSTVd can affect potato growth and development through RNA silencing, host transcriptional reprogramming, and changes in hormone and defense signaling [10,11,12,13]. The identification of 2972 genes with significant infection × region interaction effects further indicates that the two symptomatic regions exhibit distinct region-dependent molecular states. However, because samples were collected after the visible symptoms had developed, these differences cannot establish whether the observed molecular changes preceded symptom formation or arose as secondary responses to tissue damage, repair, or altered growth.

Crack regions were characterized primarily by enhanced defense and redox responses together with remodeling of the cell wall and surface barrier. Within the green module and among PSTVd-P biased response genes, *CRK2*, *RBOHA*, *PRB1-like*, *CSLG2*, *COMT*, *LAC4-like*, and *CYP86A33* were associated with receptor-kinase and ROS signaling, cell wall polysaccharide remodeling, phenylpropanoid and lignin metabolism, and barrier-lipid formation. *CRK2* regulates NADPH oxidase-mediated ROS production [36]. During wound healing in potato tubers, ROS and H_2_O_2_ participate in the construction of suberin and lignin barriers, and the associated genes are induced in a stage-dependent manner [21,37,38]. Pathogen infection of potato tubers can likewise alter ROS, hormone, and cell wall defense responses [39]. Defense and ROS-related transcriptional responses in crack regions may therefore reflect local damage and repair responses, whereas disruption of cell wall, phenylpropanoid, and barrier-lipid metabolism may be associated with loss of tissue continuity and persistence of cracking. However, some of these changes may also occur after cracks have formed.

In contrast, regions of knob-like protuberances at tuber eyes showed more pronounced reprogramming of the cell cycle, DNA replication, chromosome maintenance, and chromatin organization. *ACL5-like*, *GASA6*, *GA2OX1*, and *PILS7-like* in the black module were associated with PSTVd-E, and the infection × region interaction analysis further identified PSTVd-E biased responses of *CYCA3;1*, *CDKB1;2*, *MCM4*, *RAD51*, *RPA3B*, and *Histone H4*, which are involved in cell cycle progression, DNA replication and repair, and nucleosome organization. Release from potato tuber dormancy and reactivation of eye meristem activity are accompanied by changes in cyclin, histone, and cell cycle related genes and are regulated by CK and GA [32,40,41,42]. The present transcriptomic profile is consistent with altered cell cycle and growth-related activity in local eye tissues; however, without histological or cell proliferation measurements, an abnormal proliferative state cannot be established directly. *PILS7-like* is associated with intracellular auxin homeostasis, whereas *ACL5-like* is associated with polyamine biosynthesis and regulation of cell elongation [43,44]. These genes may represent candidate nodes linking hormones, polyamine metabolism, and local growth, but their functions require genetic and histological validation.

Phytohormone metabolomic analysis further demonstrated distinct metabolic environments in the two regions. Several CK-related metabolites increased after infection in the P region but decreased in the E region. SAG increased in both regions, with a larger increase in the P region; OPDA generally increased in P and decreased in E, whereas IBA showed the opposite pattern. The molar sum of core CK metabolites increased significantly in the P region and decreased significantly in the E region. JA-related oxylipins showed a significant infection × region interaction, but the simple effects within the individual regions were not significant; the E-region response should therefore be described as a downward trend. IAA showed a significant infection response only in the P region, whereas ABA did not change significantly in either region. These results do not support altered IAA/ABA balance as a principal hormonal feature of knob-like protuberances. Previous studies have shown that PSTVd-induced hormone and antioxidant responses are organ-dependent and that JA contributes to potato defense and symptom responses to PSTVd [16,17]. Normal activation of tuber eyes generally requires CK [32,42]; consequently, the CK decrease in the E region may reflect altered synthesis, conversion, or feedback regulation under PSTVd infection rather than indicating that reduced CK directly promotes protuberance formation.

**Figure 8:**
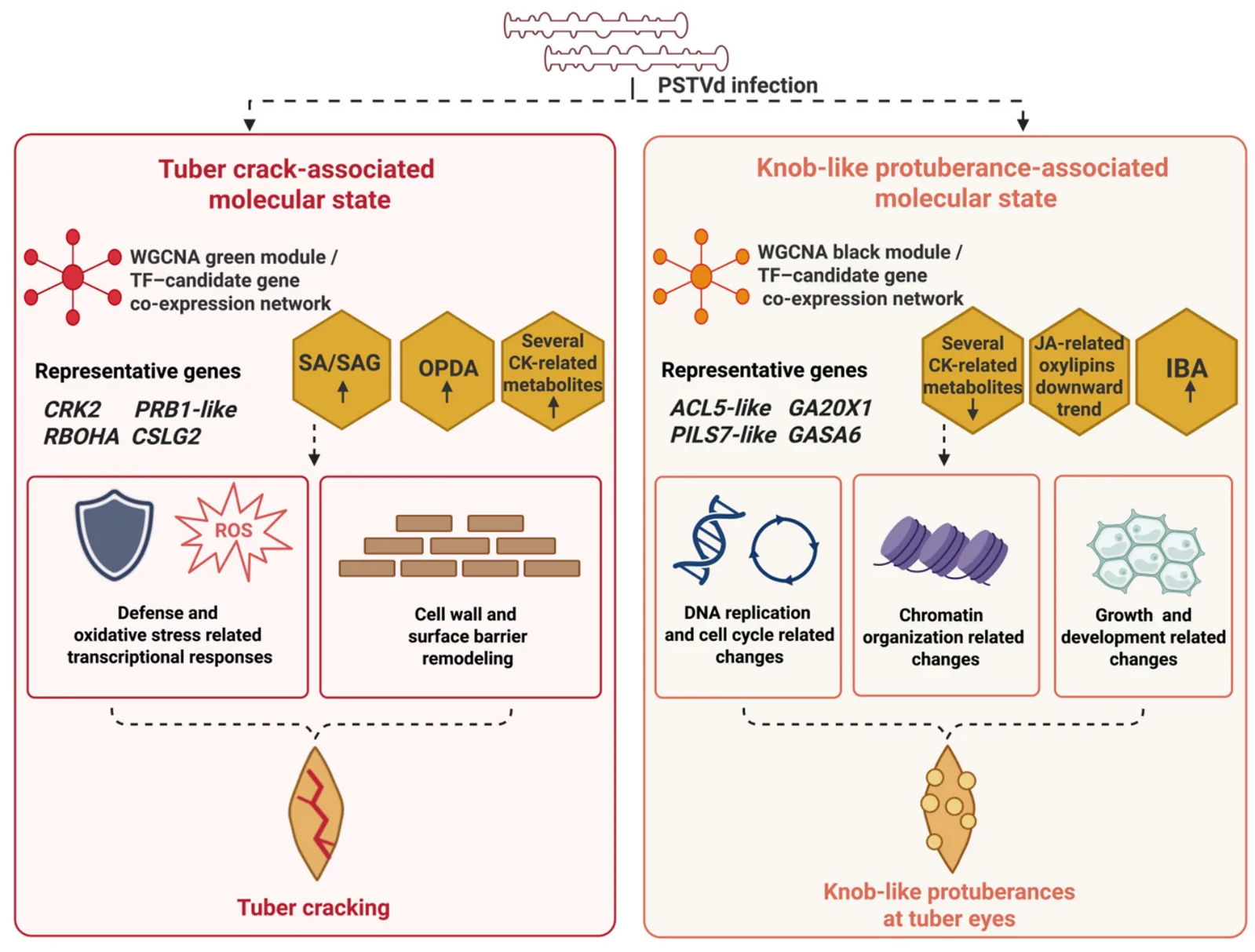
Proposed model illustrating distinct molecular states associated with PSTVd-induced tuber cracking and knob-like protuberances at tuber eyes. This model summarizes the region-associated transcriptional and phytohormone metabolic features identified in this study. The tuber crack-associated molecular state is characterized by defense and oxidative-stress-related transcriptional responses, cell wall and surface barrier remodeling, and associated hormone metabolic changes. The knob-like protuberance-associated molecular state is characterized by changes in DNA replication, cell cycle related processes, chromatin organization, growth and development-related processes, and hormone metabolism. Representative candidate genes identified from WGCNA modules are shown. The model represents an integrative working hypothesis summarizing the molecular states associated with the two PSTVd-induced symptomatic regions. Dashed arrows indicate associations rather than experimentally validated causal relationships. *CRK2*, cysteine-rich receptor-like protein kinase 2; *PRB1-like*, basic form of pathogenesis-related protein 1-like; *RBOHA*, respiratory burst oxidase homolog protein A; *CSLG2*, cellulose synthase-like protein G2; *ACL5-like*, thermospermine synthase ACAULIS5-like; *GA2OX1*, gibberellin 2-oxidase 1; *PILS7-like*, PIN-LIKES 7-like protein; *GASA6*, gibberellin-regulated protein 6.

Integrated transcriptome-phytohormone analysis showed that cell wall, ROS, and defense-related genes associated with cracking were generally positively correlated with CK, SA/SAG, and OPDA-related indices. In contrast, genes associated with knob-like protuberances were mainly negatively correlated with TRA, the molar sum of core CK metabolites, and cZROG. Together with the differential-expression, co-expression, and phytohormone results, these exploratory correlations are consistent with distinct region-associated molecular states in the two symptomatic regions, as summarized schematically in Figure 8. Nevertheless, the network was based on only 12 samples, and some strong correlations may have been driven by overall separation among groups; therefore, direct regulatory relationships cannot be inferred.

Overall, crack regions were characterized mainly by defense and ROS-related responses, remodeling of the cell wall and surface barrier, and accumulation of several CK-related metabolites, SA/SAG, and OPDA. Regions of knob-like protuberances at tuber eyes were characterized primarily by reprogramming of the cell cycle, DNA replication, and chromatin organization, together with decreases in several CK-related metabolites, a downward trend in JA-related oxylipins, and increased IBA. Because samples were collected at 80 dpi after symptoms had developed, the present results primarily describe molecular states associated with the two symptomatic regions and cannot establish whether the observed changes preceded or followed symptom formation. WGCNA and correlation networks should also be regarded as exploratory. Because the present study examined only the potato cultivar ‘Kexin 18’ infected with a single severe PSTVd strain, the molecular states identified here should be interpreted within the context of this host genotype-viroid strain combination and should not be generalized to other potato cultivars or PSTVd variants without further validation. To distinguish molecular changes that precede symptom development from those associated with symptom progression or maintenance, future time-course studies should include sampling before visible symptom appearance, during the early stages of crack or protuberance development, and after the symptoms are fully established. Histological and cell proliferation analyses, together with functional validation of candidate genes, will also be required to evaluate causal relationships.

## 4. Materials and Methods

### 4.1. Plant Material and Growth Conditions

In vitro plantlets of the potato cultivar‘Kexin 18’(K18), maintained by the Chongqing Key Laboratory of Potato Biology and Genetic Breeding, were used in this study. Stem segments were aseptically propagated on solid Murashige and Skoog (MS) medium [45] at 22 °C under a 16 h light/8 h dark photoperiod. After the culture medium was removed from the roots, rooted plantlets were pre-acclimated with their roots immersed in distilled water for 48 h and then transplanted into a peat:vermiculite substrate (3:1, *v*/*v*). The plants were covered with plastic film for 48 h to maintain humidity and were subsequently grown to the five-leaf stage for PSTVd inoculation.

### 4.2. PSTVd Inoculation

*Agrobacterium tumefaciens* carrying the pCAMBIA1300::35S-PSTVd-S plasmid was inoculated into YEB medium containing kanamycin (100 μg/mL) and rifampicin (50 μg/mL) and cultured overnight at 28 °C and 200 rpm. The culture was transferred to 50 mL of fresh YEB medium and incubated at 28 °C and 250 rpm for approximately 6 h until OD_600_ reached approximately 0.5. Cells were collected by centrifugation at 5000 rpm for 10 min, resuspended in MES infiltration buffer, and incubated in darkness at 28 °C for 3 h. Leaves were infiltrated with a needleless syringe, with two to three leaves inoculated per plant. After inoculation, plants were maintained in darkness at 25 °C for 48 h and then transferred to 25 °C under a 16 h light/8 h dark photoperiod. Mock plants were treated identically with *Agrobacterium* carrying the empty vector. PSTVd-S is a severe strain (GenBank accession X58388.1).

### 4.3. Sample Collection for Transcriptomic and Phytohormone Metabolomic Analyses

A total of 30 plants were inoculated with PSTVd, and 30 plants were mock-inoculated. Prior to tuber sampling, leaf samples from individual plants were analyzed by RT-qPCR to confirm PSTVd infection status. At 80 days post-inoculation, four tissue types were collected from tubers of nine plants per treatment for transcriptomic and phytohormone metabolomic analyses: (1) Mock-P, smooth surface regions of mock-inoculated tubers; (2) Mock-E, normal eye tissues of mock-inoculated tubers; (3) PSTVd-P, crack regions of PSTVd-infected tubers; and (4) PSTVd-E, regions of knob-like protuberances at tuber eyes of PSTVd-infected tubers. For both the Mock and PSTVd treatments, three independent biological replicates were established. Each replicate consisted of tuber tissues pooled in equal amounts from three independent plants, with no plants shared among replicates. Within each replicate, tissues from the P and E regions were collected separately from the same set of three plants and pooled by region. Thus, P and E samples with the same replicate number were derived from the same plant pool and were treated as paired samples. This design yielded four groups: Mock-P, Mock-E, PSTVd-P, and PSTVd-E, with three biological replicates per group, for a total of 12 samples. Samples were immediately frozen in liquid nitrogen and stored at −80 °C. RNA sequencing was performed by Gene Denovo Biotechnology Co., Ltd. (Guangzhou, China), and phytohormone metabolomic analysis was performed by Metware Biotechnology Co., Ltd. (Wuhan, China).

### 4.4. PSTVd Quantification, Candidate-Gene RT-qPCR, and Sanger Sequencing

Total RNA was extracted using the FastPure Plant Total RNA Isolation Kit (RC411, Vazyme, Nanjing, China). For all samples, 1 μg of total RNA was reverse-transcribed using Hifair^®^ III 1st Strand cDNA Synthesis SuperMix for qPCR (Yeasen, Shanghai, China). All real-time quantitative PCR assays were performed using a CFX Opus 96 system (Bio-Rad, Hercules, CA, USA).

Following reverse transcription, PSTVd cDNA copy equivalents were estimated by probe-based qPCR using a FAM/TAMRA-labeled probe and Premix Ex Taq™ (Probe qPCR, RR390A, TaKaRa, Kusatsu, Shiga, Japan). Reactions were performed in a total volume of 10 μL under the following conditions: 95 °C for 10 min, followed by 45 cycles of 95 °C for 10 s, 60 °C for 50 s, and 72 °C for 1 s. A recombinant plasmid containing the PSTVd sequence was used to generate the standard curve, with each concentration assayed in triplicate. The regression equation was Ct = −3.1541 log_10_(copy concentration) + 39.033 (R^2^ = 0.9997; PCR efficiency = 107.5%). Late amplification signals were observed in the mock-inoculated samples at Ct 30.57–31.38. Reactions with no detectable amplification were considered negative, whereas these late signals were treated as background and excluded from cDNA copy-equivalent estimation. No-RT controls were included and showed no detectable amplification. Because the plasmid standard did not undergo reverse transcription, the values are reported as PSTVd cDNA copy equivalents rather than absolute PSTVd RNA copy numbers. Because the same amount of total RNA (1 μg) was used for reverse transcription of each sample, the resulting estimates represent PSTVd cDNA copy equivalents obtained from equal total-RNA inputs and were not normalized to tissue mass, cell number, or a host reference gene.

Candidate genes were relatively quantified using TB Green^®^ Premix Ex Taq™ II (TaKaRa, Kusatsu, Shiga, Japan). The amplification program comprised 95 °C for 30 s, followed by 39 cycles of 95 °C for 5 s, 60 °C for 10 s, and 72 °C for 30 s, followed by melting-curve analysis. *StEF1α* was used as the reference gene [46], Mock-P served as the calibrator, and relative expression was calculated using the 2^−ΔΔCt^ method [47]. The amplification efficiencies of all primer pairs used for candidate-gene RT-qPCR ranged from 90% to 105%. The Ct values of *StEF1α* were stable across the four sample groups, ranging from 21.15 to 21.70, with group means ranging from 21.29 to 21.53; no significant difference was detected among groups (one-way ANOVA, *p* = 0.230). Three biological replicates were analyzed per group, with three technical replicates per biological replicate. Full-length PSTVd amplicons were generated from three biological replicates each of PSTVd-P and PSTVd-E using PSTVd-Seq1 and PSTVd-Seq2 and were subjected to bidirectional Sanger sequencing with the same primers. Consensus sequences were compared with the inoculated PSTVd-S sequence (GenBank accession X58388.1). Primers and probes are listed in Table S9.

### 4.5. RNA Sequencing and Bioinformatics Analysis

Total RNA was extracted from each sample using TRIzol reagent. RNA quality and integrity were assessed using an Agilent 2100 Bioanalyzer (Agilent, Santa Clara, CA, USA) and agarose gel electrophoresis. RNA samples that met the quality criteria were used for cDNA library construction and high-throughput sequencing on the Illumina NovaSeq 6000 platform.

Raw sequencing data were quality-filtered using fastp (v0.18.0) [48]. Reads containing adaptor sequences, more than 10% ambiguous bases, continuous poly(A) tracts, or low-quality bases (Q ≤ 20) accounting for more than 50% of the read length were removed to obtain high-quality clean reads. Clean reads were aligned to the potato DM v6.1 reference genome (http://spuddb.uga.edu/) using HISAT2 (v2.2.4) [49,50]. Gene expression was quantified with RSEM [51] and expressed as fragments per kilobase of transcript per million mapped reads (FPKM).

Differential expression analysis was performed on the raw read-count matrix using DESeq2 [52]. For the within-treatment regional comparisons, Mock-P vs. Mock-E and PSTVd-P vs. PSTVd-E, the paired structure was accounted for by including biological plant-pool identity as a blocking factor (design: ~pool + region). For the infection comparisons, Mock-P vs. PSTVd-P and Mock-E vs. PSTVd-E, the Mock and PSTVd samples were derived from independent plant pools and were analyzed using a design of ~infection. Differentially expressed genes (DEGs) were identified using thresholds of FDR < 0.05 and |log_2_(fold change)| > 1. To identify region-dependent transcriptional responses to PSTVd infection while accounting for the paired-pool structure within each infection treatment, an infection-by-region interaction analysis was performed using DESeq2. The full model was specified as ~infection + infection:pool_n + infection:region, whereas the reduced model was specified as ~infection + infection:pool_n + region. Here, pool_n was treated as a categorical factor nested within infection treatment, representing the matched P- and E-region samples derived from the same biological plant pool. The significance of the infection-by-region interaction was evaluated using a likelihood ratio test. The signed interaction contrast was defined as (PSTVd-E − PSTVd-P) − (Mock-E − Mock-P), which is algebraically equivalent to [PSTVd-E − Mock-E] − [PSTVd-P − Mock-P]. Positive interaction coefficients indicated relatively stronger or more positive infection responses in the E region, whereas negative coefficients indicated relatively stronger or more positive infection responses in the P region. Genes with an LRT FDR < 0.05 and an absolute estimated interaction log_2_ fold change ≥ 1 were considered to exhibit significant infection-by-region interaction effects.

Gene Ontology (GO; http://geneontology.org/) and Kyoto Encyclopedia of Genes and Genomes (KEGG; https://www.genome.jp/kegg/) databases were used for functional and pathway enrichment analyses of DEGs [53,54], and the results were visualized using the OmicShare platform (https://www.omicshare.com/). A targeted preranked gene set enrichment analysis (GSEA) was additionally performed using genes ranked by the DESeq2 Wald statistic from the infection × region interaction analysis [55]. Gene sets containing 10-500 genes were evaluated using 5000 permutations, and *p* values were adjusted separately within the predefined GO and KEGG panels using the Benjamini–Hochberg procedure, with q < 0.05 considered significant.

### 4.6. Weighted Gene Co-Expression Network Analysis

A gene-expression matrix from the 12 samples was used for WGCNA after removal of lowly expressed genes with counts ≤ 2 in all samples [56]. A soft-thresholding power of β = 14 was selected according to the scale-free topology fit index and mean connectivity. The minimum module size was set to 100, and co-expression modules were identified using the topological overlap matrix, hierarchical clustering, and dynamic tree cutting [57].

The four sample types were encoded as binary traits, and Pearson correlation coefficients were calculated between module eigengenes and each sample type. Module–trait relationships were evaluated descriptively, and modules were ranked according to correlation strength rather than statistical significance. The modules showing the strongest correlations with PSTVd-P and PSTVd-E were designated as candidate modules associated with tuber cracking and knob-like protuberances at tuber eyes, respectively. Candidate genes were first identified at the intersection of DEGs and the corresponding phenotype-associated module and ranked by intramodular connectivity. Functional annotation and phenotype relevance were then considered, and functionally redundant members of the same gene family were excluded to obtain representative candidate genes and transcription factors. Functional annotation and phenotype relevance were used only to prioritize candidates after statistical filtering and connectivity ranking and were not treated as independent statistical evidence of regulatory importance. Intramodular connectivity, connectivity rankings, and selection criteria are provided in Tables S4 and S5. TF-candidate gene co-expression networks were visualized using Cytoscape [58]. Because the WGCNA was based on only 12 RNA-seq samples, the resulting modules and candidate genes were used primarily to explore phenotype-associated co-expression patterns.

### 4.7. Targeted Quantification of Phytohormones

Phytohormone measurements were performed by Metware Biotechnology Co., Ltd. (Wuhan, China). Fifty milligrams of tuber tissue ground in liquid nitrogen was mixed with 10 μL of a mixed internal-standard solution (100 ng/mL) and 1 mL of methanol/water/formic acid extraction solution (15:4:1, *v*/*v*/*v*). Samples were vortexed for 10 min and centrifuged at 12,000 rpm for 5 min at 4 °C. The supernatant was collected and evaporated to dryness, reconstituted in 100 μL of 80% aqueous methanol, filtered through a 0.22 μm membrane, and subjected to LC-MS/MS analysis.

Samples were analyzed using an ExionLC™ AD ultrahigh-performance liquid chromatography system coupled to a QTRAP^®^ 6500+ tandem mass spectrometer. Data were acquired in positive- and negative-ion electrospray ionization modes using scheduled multiple-reaction monitoring. Mass-spectrometric data were acquired with Analyst 1.6.3, and chromatographic peaks were integrated and quantified using MultiQuant 3.0.3. Hormones and related metabolites were quantified using authentic standards and internal standards. Standard curves were generated using the concentration ratio of each standard to its internal standard as the x-axis and the corresponding peak-area ratio as the y-axis. Metabolite contents were expressed as ng g^−1^ fresh weight (FW) [59]. For pairwise comparisons, metabolites exceeding the predefined fold-change threshold (fold change ≥ 2 or ≤0.5) were identified, and KEGG was used for pathway annotation and exploratory enrichment analysis [54].

Region-dependent changes in individual hormone metabolites were analyzed after log_2_ transformation of positive quantified values. Because matched P- and E-region samples were derived from the same biological plant pool, infection × region interactions were evaluated using replicate-wise regional contrasts calculated as log_2_(E) − log_2_(P), with the interaction defined as the difference in these contrasts between PSTVd and Mock treatments. Regional contrasts and simple PSTVd-versus-Mock effects within the P and E regions were evaluated using two-sided Welch’s *t*-test. *p* values were adjusted separately for the interaction and simple-effect test families using the Benjamini–Hochberg procedure. To compare the overall states of different hormone-metabolic pathways, relevant metabolite contents were converted to pmol g^−1^ FW, and predefined molar sums and molar ratios of hormone metabolites were calculated within each biological replicate. The components, formulas, and results for each index are provided in Table S7. Molar ratios were log_2_-transformed before statistical analysis. The same interaction and simple-effect testing procedures described above were applied to these derived hormone indices.

### 4.8. Integrated Transcriptomic and Phytohormone Metabolomic Analysis

Representative genes associated with tuber cracking and knob-like protuberances at tuber eyes were selected by integrating differential expression, WGCNA, and infection × region interaction results. These genes were jointly analyzed with key hormone metabolites, molar sums of hormone metabolites, and molar ratios. Pearson correlation coefficients and two-sided *p* values were calculated using log_2_(FPKM+1)-transformed gene-expression values and log_2_-transformed hormone-related indices from the 12 matched biological samples. A total of 555 correlations were tested between 37 candidate genes and 15 hormone-related indices. The *p* values from all 555 tests were adjusted together using the Benjamini–Hochberg false discovery rate (BH-FDR) procedure. Correlation heatmaps display Pearson coefficients and BH-FDR-adjusted significance levels, with q < 0.05 and q < 0.01 indicating significant and highly significant correlations, respectively. Gene-hormone association networks for tuber cracking and knob-like protuberances at tuber eyes were constructed using thresholds of |r| ≥ 0.80 and BH-FDR-adjusted q < 0.05. Network nodes represent candidate genes, hormone metabolites, molar sums, or molar ratios; red and blue edges indicate positive and negative correlations, respectively, and edge width represents correlation strength. Correlation heatmaps and network diagrams were generated in R version 4.6.1.

### 4.9. Statistical Analysis and Figure Preparation

Unless otherwise stated, three biological replicates were analyzed per group, and data are presented as the mean ± SD or SEM. Statistical analysis of RT-qPCR data was based on ΔCt values from biological replicates. Mock and PSTVd samples within the same region were compared using two-sided Welch’s *t*-test, whereas P and E regions within the same treatment were compared using two-sided paired *t*-tests. The RT-qPCR analysis of the six selected genes was used to assess concordance with the corresponding RNA-seq expression patterns and was treated as exploratory; therefore, *p* values were not adjusted for multiple testing. Hormone data were analyzed as described in Section 4.7. Gene-hormone correlations were assessed using two-sided Pearson tests, and the *p* values from all 555 gene–hormone tests were adjusted together using the Benjamini–Hochberg false discovery rate procedure; only associations with |r| ≥ 0.80 and BH-FDR-adjusted q < 0.05 were retained in the networks. GO and KEGG enrichment of transcriptomic data was considered significant at q < 0.05, whereas KEGG enrichment of hormone metabolites exceeding the fold-change threshold was treated as exploratory. Data analyses and figure preparation were conducted primarily using R version 4.6.1, Cytoscape v3.9.1, and the OmicShare platform (www.omicshare.com).

## 5. Conclusions

In PSTVd-infected tubers sampled after symptom development, tuber crack regions and regions with knob-like protuberances at tuber eyes exhibited distinct region-dependent transcriptomic and phytohormone metabolic states. Crack regions were characterized mainly by defense and ROS-related transcriptional responses, remodeling of the cell wall and surface barrier, and accumulation of several CK-related metabolites, SA/SAG, and OPDA. In contrast, regions with knob-like protuberances at tuber eyes were characterized by transcriptional changes related to DNA replication, cell-cycle regulation, chromosome maintenance, and chromatin organization, together with decreases in several CK-related metabolites, a downward trend in JA-related oxylipins, and increased IBA. Together, these findings characterize distinct molecular states associated with the two symptomatic regions in the sampled tubers, while the temporal and causal relationships between these molecular changes and symptom development remain unresolved.

## Data Availability

The raw RNA-sequencing data generated in this study have been deposited in the NCBI Sequence Read Archive (SRA) under BioProject PRJNA1499971. The four SRR accessions correspond to the four experimental groups: Mock-P (SRR39832580), Mock-E (SRR39832579), PSTVd-P (SRR39832578), and PSTVd-E (SRR39832577). In the current SRA deposition, the three independently prepared biological-replicate libraries from each experimental group are associated with a single SRR accession. The original submitted FASTQ files remain separate and identifiable by replicate-specific filenames, with one paired-end R1/R2 file pair corresponding to each biological library, giving 12 biological libraries in total. The complete mapping among experimental groups, biological replicates, BioSample accessions, SRR accessions, and original FASTQ filenames is provided in Supplementary Table S10.

## Supplementary Materials

The following supporting information can be downloaded at https://www.mdpi.com/article/10.3390/plants15182842/s1, Figure S1: Pearson correlation analysis among RNA-seq samples; Figure S2: WGCNA network construction, module detection, and module-trait relationships; Figure S3: Bidirectional preranked GSEA of region-dependent responses following PSTVd infection; Figure S4: Overall distribution and KEGG enrichment analysis of hormone metabolites exceeding the predefined fold-change threshold; Figure S5: Phytohormone changes in different tuber regions following PSTVd infection; Figure S6: Relative expression levels of selected genes in distinct phenotypic regions of PSTVd-infected tubers determined by RT-qPCR. Table S1: Summary of RNA-seq data quality and mapping statistics; Table S2: Differentially expressed genes identified in the four pairwise comparisons; Table S3: Complete GO and KEGG enrichment results for the crack- and knob-like protuberance-associated comparisons; Table S4: WGCNA module-trait relationships, candidate genes, transcription factors, and TF-candidate gene co-expression relationships associated with tuber cracking; Table S5: WGCNA module-trait relationships, candidate genes, transcription factors, and TF-candidate gene co-expression relationships associated with knob-like protuberances at tuber eyes; Table S6: Infection-by-region interaction genes, GO/KEGG enrichment results, and representative PSTVd-E and PSTVd-P biased response genes used in Figure 5; Table S7: Absolute hormone metabolite contents, derived molar sums and ratios, and infection × region interaction and simple-effect analyses; Table S8: Pearson correlations between phenotype-associated genes and hormone-related features; Table S9: Primer and probe sequences used for PSTVd quantification, candidate-gene RT-qPCR, and full-length PSTVd amplification and Sanger sequencing; Table S10: RNA-seq biological replicate and NCBI SRA file mapping; Supplementary File S1: Bidirectional Sanger sequencing results for PSTVd from the PSTVd-P and PSTVd-E regions.
