# Supplementary material for: Integrated Transcriptomic and Phytohormone Metabolomic Analyses Reveal Distinct Molecular States Associated with PSTVd-Induced Potato Tuber Cracking and Knob-like Protuberances at Tuber Eyes
Source: Plants (Basel). 2026 Sep 17;15(18):2842. doi: 10.3390/plants15182842 (PMC13610134; doi:10.3390/plants15182842)
Supplement: Supplementary file 1 [file plants-15-02842-s001.zip › Supplementary Figures.pdf]

Supplementary File

Integrated Transcriptomic and Phytohormone Metabolomic Analyses Reveal Distinct Molecular States Associated with PSTVd-Induced Potato Tuber Cracking and Knob-like Protuberances at Tuber Eyes

Guangyan Li [1,2,3,†], Wajahat Hussain [1,2,3,†], Ting Pan [1,2,3], Yingyan Chen [1,2,3], Jiaqi Li [1,2,3], Yunxia Zeng [1,2,3], Yonghong Zhou [1,2,3,*] and Dianqiu Lv [1,2,3,*]

1 College of Agronomy and Biotechnology, Southwest University, Chongqing 400715, China; lihayy616@163.com (G.L.); wajahat7253@yahoo.com (W.H.); 15736270797@163.com (T.P.); 13118924035@163.com (Y.C.); 15136826253@163.com (J.L.); 17341868521@163.com (Y.Z.)
2 Integrative Science Center of Germplasm Creation in Western China (Chongqing) Science City, Southwest University, Chongqing 400715, China
3 Chongqing Key Laboratory of Potato Biology and Genetic Breeding, Southwest University, Chongqing 400715, China
* Correspondence: zhou2017@swu.edu.cn (Y.Z.); smallpotatoes@126.com (D.L.)
† These authors contributed equally to this work.

Supplementary Figures

Figure S1. Pearson correlation analysis among RNA-seq samples

Figure S2. WGCNA network construction, module detection, and module–trait relationships

Figure S3. Bidirectional preranked GSEA of region-dependent responses following PSTVd infection

Figure S4. Overall distribution and KEGG enrichment analysis of hormone metabolites exceeding the predefined fold-change threshold

Figure S5. Phytohormone changes in different tuber regions following PSTVd infection

Figure S6. Relative expression levels of selected genes in distinct phenotypic regions of PSTVd-infected tubers determined by RT-qPCR

Academic Editors: Luca Ferretti and Anna Taglienti

Received: 1 August 2026

Revised: 10 September 2026

Accepted: 14 September 2026

Published: date

Copyright: © 2026 by the authors. Submitted for possible open access publication under the terms and conditions of the Creative Commons Attribution (CC BY) license.

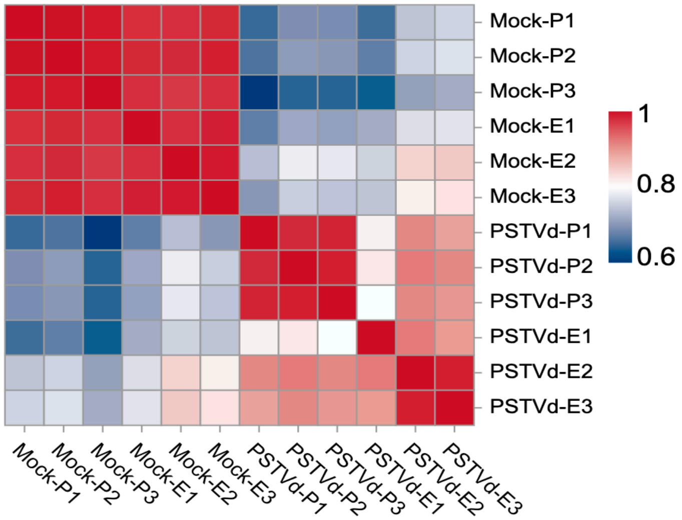

Figure S1. Pearson correlation analysis among RNA-seq samples. Pearson correlation coefficients among the 12 RNA-seq samples were calculated using the global gene-expression profiles. Colors indicate correlation strength, with red and blue denoting higher and lower correlations, respectively. Mock-P and Mock-E represent smooth surface regions and normal eye tissues, respectively, from mock-inoculated tubers; PSTVd-P and PSTVd-E represent crack regions and regions bearing knob-like protuberances at tuber eyes, respectively, from PSTVd-infected tubers. Each group comprised three biological replicates.

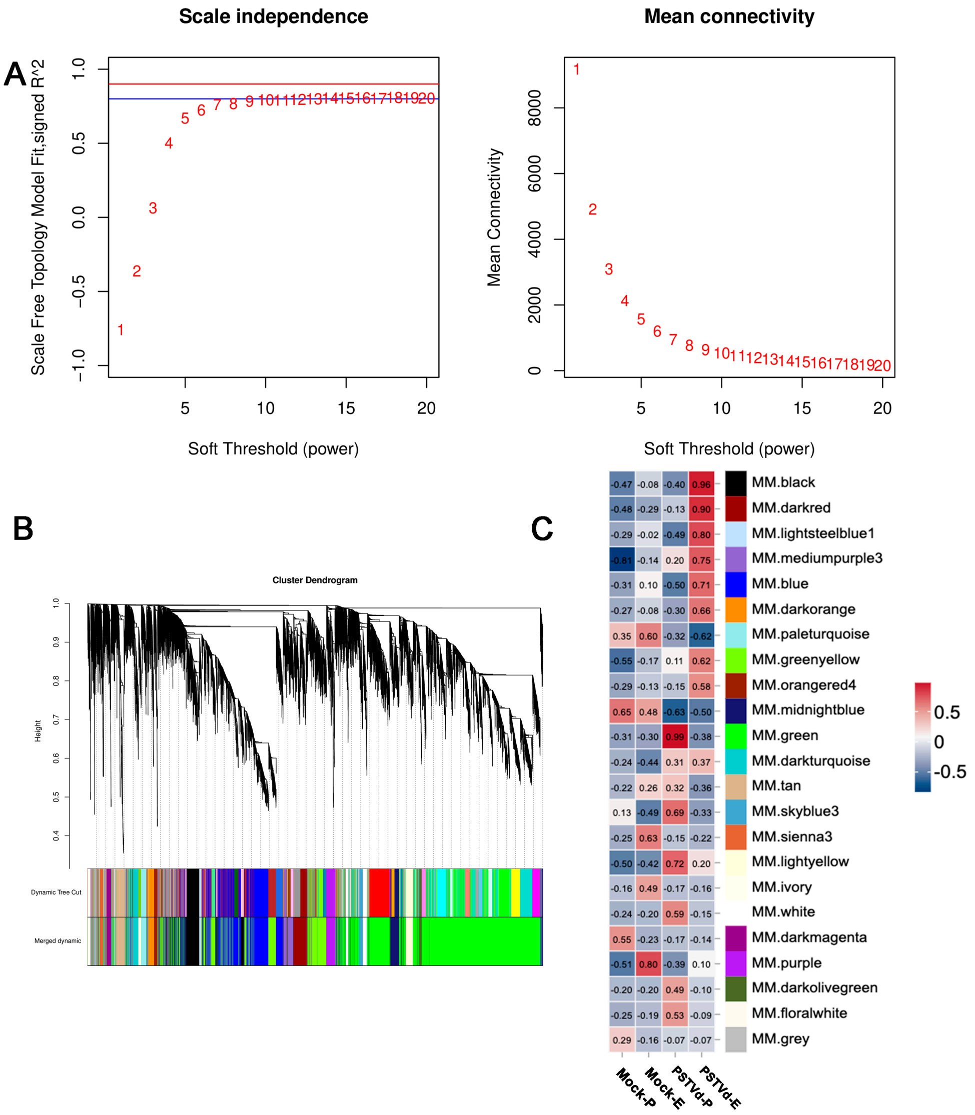

Figure S2. WGCNA network construction, module detection, and module–trait relationships. (A) Soft-thresholding power analysis. The left panel shows the scale-free topology fit index at different soft-thresholding powers, and the right panel shows the corresponding mean connectivity. Based on the balance between scale-free topology fit and mean connectivity, $\beta = 14$ was selected for network construction. (B) Gene hierarchical clustering dendrogram and module assignment. Different colors indicate distinct co-expression modules. (C) Correlations between module eigengenes and the four sample types. Values in the heatmap are Pearson correlation coefficients, and colors indicate the direction and magnitude of the correlations. Mock-P and Mock-E represent smooth surface regions and normal eye tissues, respectively, from mock-inoculated tubers; PSTVd-P and PSTVd-E represent crack regions and regions bearing knob-like protuberances at tuber eyes, respectively, from PSTVd-infected tubers.

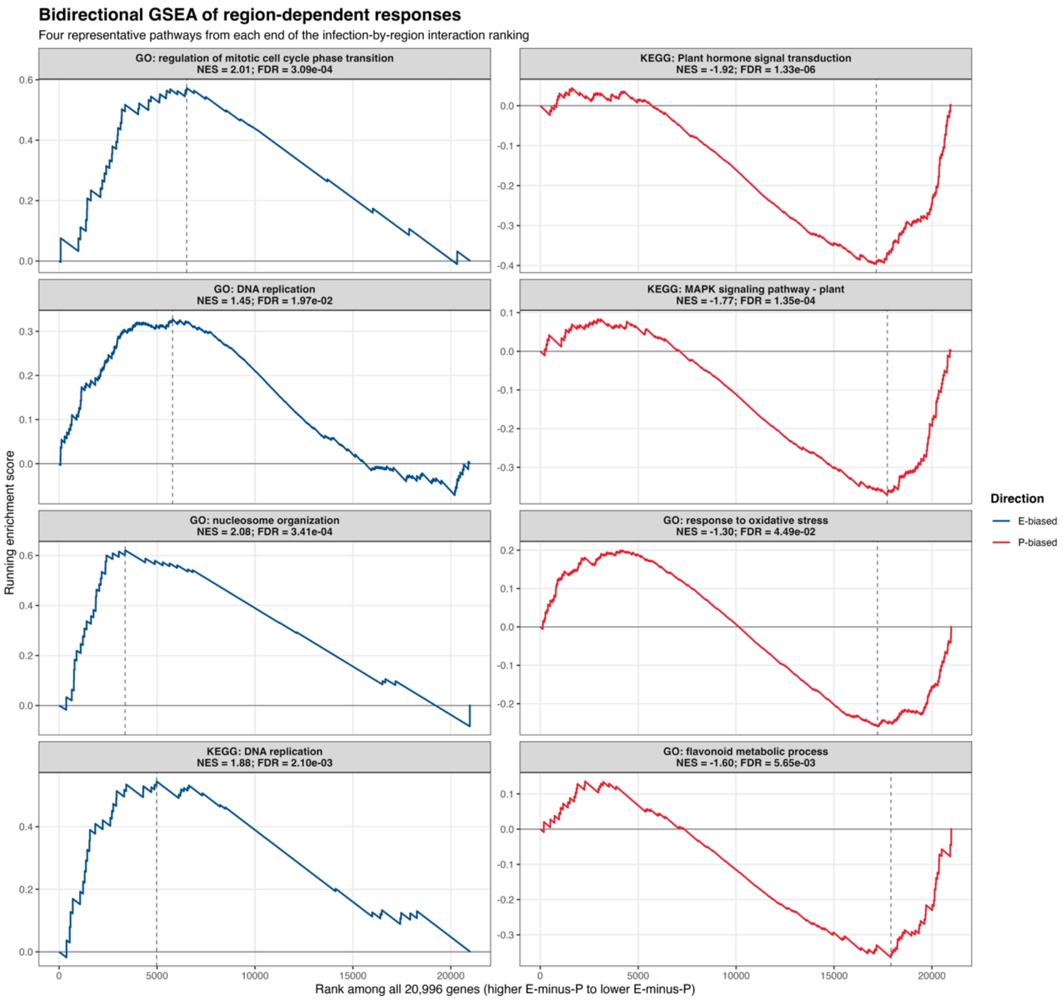

Figure S3. Bidirectional preranked GSEA of region-dependent responses following PSTVd infection. Genes were ranked by the infection × region interaction statistic [PSTVd-E − Mock-E] − [PSTVd-P − Mock-P]. Blue curves in the left column indicate enrichment toward PSTVd-E biased responses, whereas red curves in the right column indicate enrichment toward PSTVd-P biased responses. Dashed vertical lines mark the positions of the peak enrichment scores. The normalized enrichment score (NES) and Benjamini–Hochberg-adjusted false discovery rate (FDR) are shown in each panel.

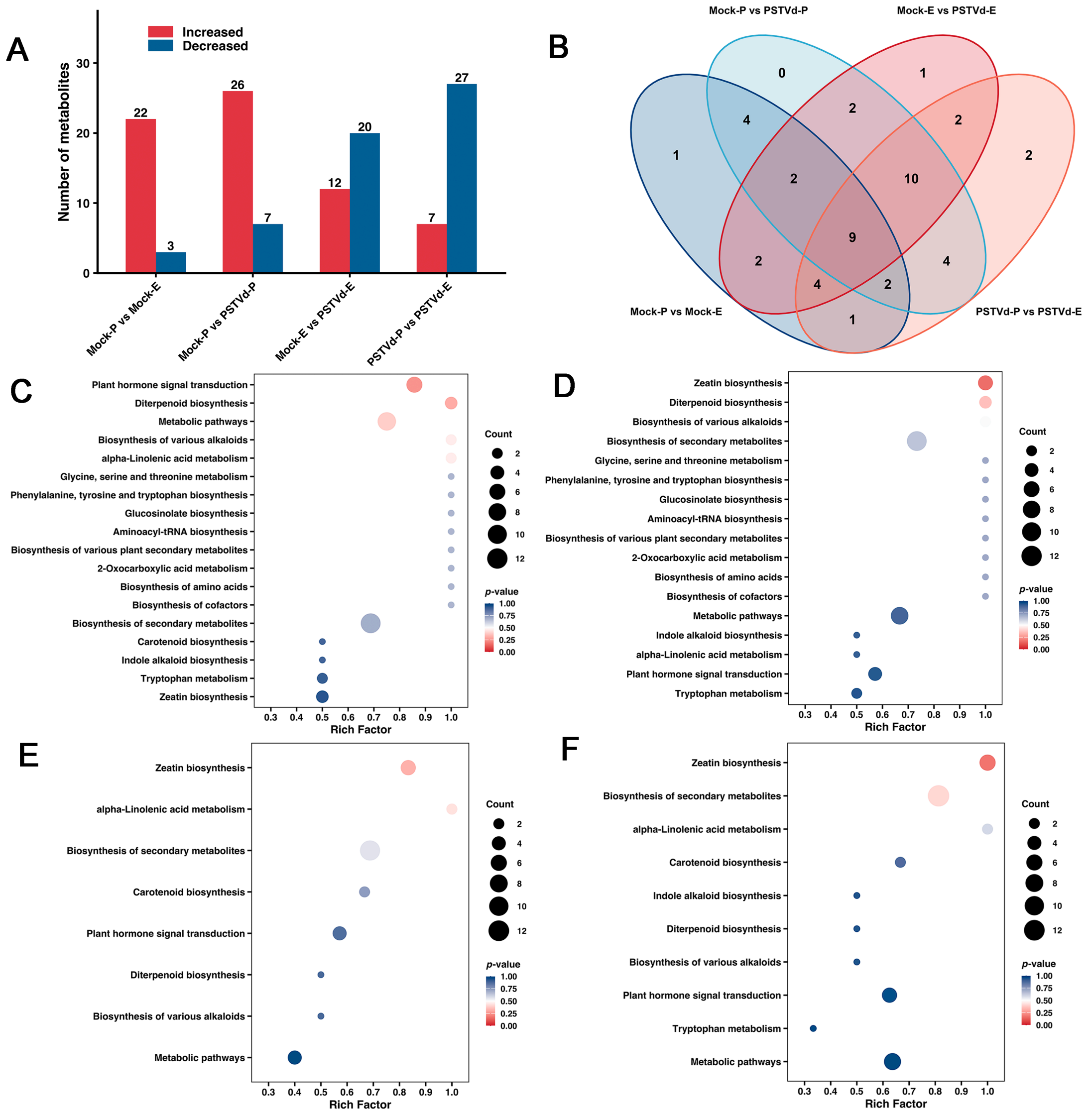

Figure S4. Overall distribution and KEGG enrichment analysis of hormone metabolites exceeding the predefined fold-change threshold. (A) Numbers of increased and decreased hormone metabolites exceeding the predefined fold-change threshold in the four pairwise comparisons. Red and blue bars indicate increased and decreased metabolites, respectively. (B) Venn diagram showing comparison-specific and shared metabolites exceeding the predefined fold-change threshold. (C–F) Exploratory KEGG pathway enrichment analysis of metabolites exceeding the predefined fold-change threshold in Mock-P versus Mock-E (C), Mock-P versus PSTVd-P (D), Mock-E versus PSTVd-E (E), and PSTVd-P versus PSTVd-E (F). Bubble size indicates the number of metabolites assigned to each pathway, color represents the *p*-value, and the rich factor is the proportion of enriched metabolites relative to all metabolites annotated to the corresponding pathway.

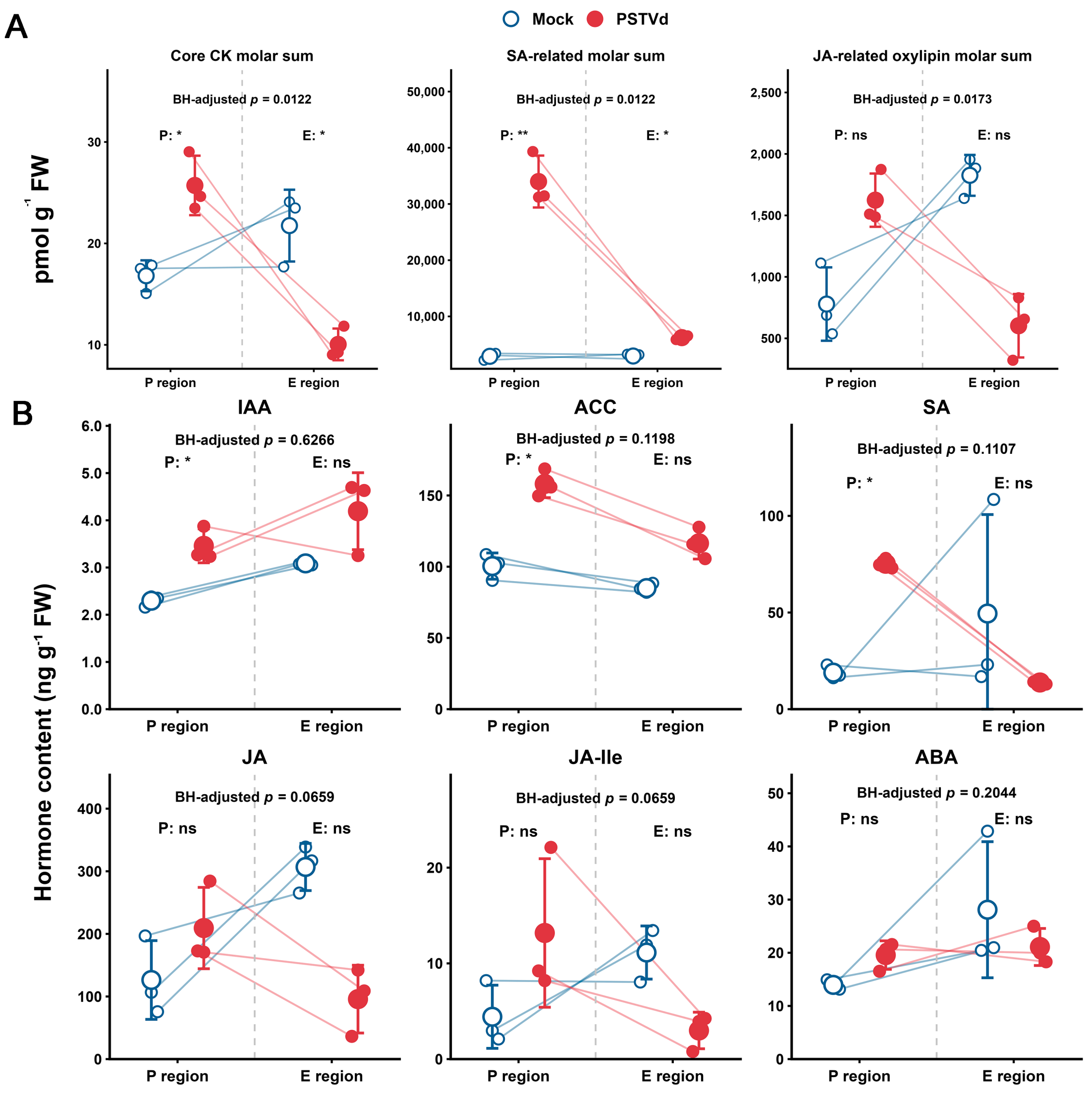

Figure S5. Phytohormone changes in different tuber regions following PSTVd infection. (A) Molar sums of core CK metabolites, SA-related metabolites, and JA-related oxylipins. (B) Contents of IAA, ACC, SA, JA, JA-Ile, and ABA. Small points represent the three biological replicates, large points represent group means, and error bars indicate SD. Lines connect paired P- and E-region samples within the same treatment. Open blue and filled red circles indicate Mock and PSTVd treatments, respectively. P and E denote the surface/crack-associated and eye/protuberance-associated regions, respectively. Symbols following P and E indicate the simple effects of PSTVd versus Mock within the corresponding region. BH-adjusted p values shown above each panel correspond to the infection × region interaction. p values were adjusted for multiple testing using the Benjamini-Hochberg procedure. n = 3; *BH-adjusted $p < 0.05$, **BH-adjusted $p < 0.01$, and ns, BH-adjusted $p \geq 0.05$.

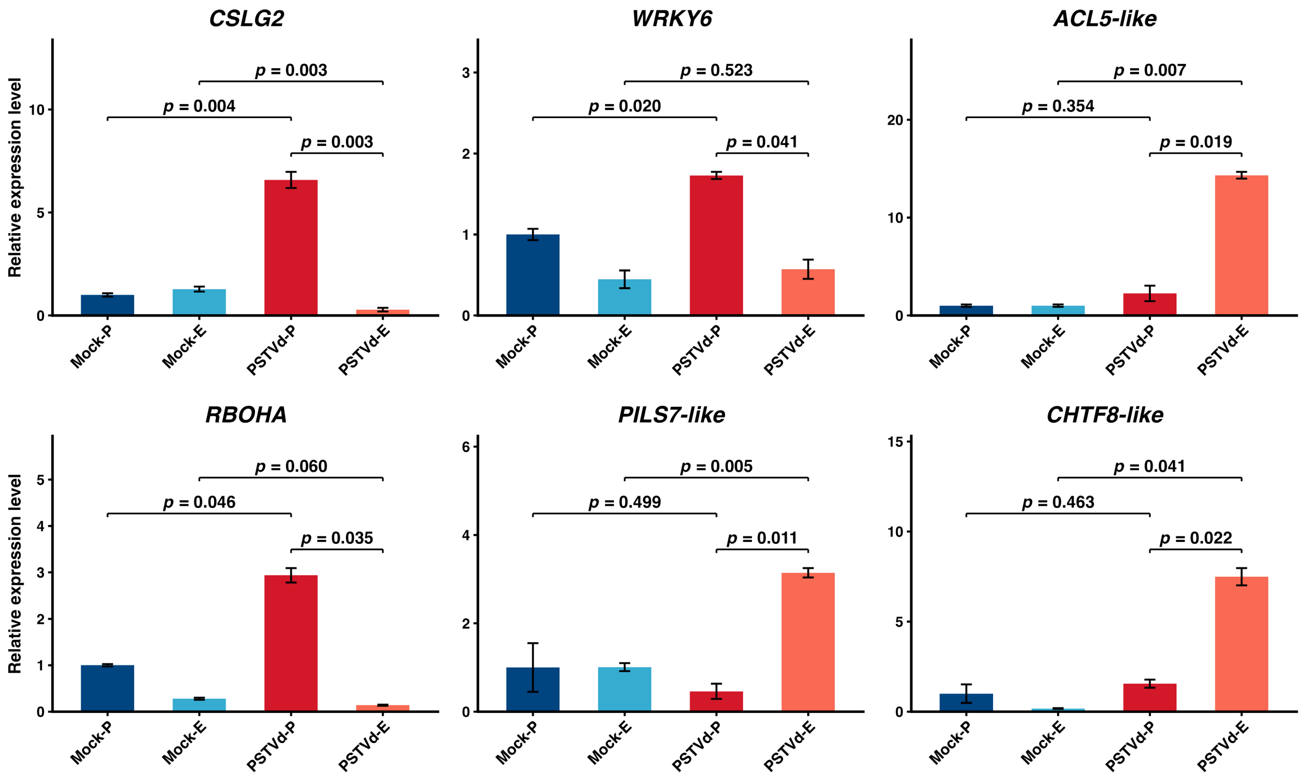

Figure S6. Relative expression levels of selected genes in distinct phenotypic regions of PSTVd-infected tubers determined by RT-qPCR. RT-qPCR analysis of the crack-associated genes *CSLG2*, *WRKY6*, and *RBOHA* and the knob-like protuberance-associated genes *ACL5-like*, *PILS7-like*, and *CHTF8-like* in Mock-P, Mock-E, PSTVd-P, and PSTVd-E samples. Bars show mean ± SEM. Statistical comparisons were performed between Mock-P and PSTVd-P, Mock-E and PSTVd-E, and PSTVd-P and PSTVd-E, with exact *p* values indicated above the corresponding comparisons. P and E denote the surface/crack-associated and eye/protuberance-associated regions, respectively. Relative expression was normalized to *StEF1α*, calibrated to Mock-P, and calculated using the $2^{-\Delta\Delta Ct}$ method.
