# Supplementary material for: Integrated Transcriptomic and Phytohormone Metabolomic Analyses Reveal Distinct Molecular States Associated with PSTVd-Induced Potato Tuber Cracking and Knob-like Protuberances at Tuber Eyes
Source: Plants (Basel). 2026 Sep 17;15(18):2842. doi: 10.3390/plants15182842 (PMC13610134; doi:10.3390/plants15182842)

Supplementary File S1. PSTVd Sanger Sequencing Results

To examine whether the two symptomatic tuber regions contained different dominant PSTVd sequence variants, PSTVd amplicons from three biological replicates each of PSTVd-E and PSTVd-P were subjected to bidirectional Sanger sequencing.

Sequencing primers

**PSTVd-Seq1 (provider label 227)**	`5'-AGGAACCAACTGCGGTTCCAA-3'`
**PSTVd-Seq2 (provider label 228)**	`5'-CGGAACTAAACTCGTGGTTC-3'`

Sample identities

PSTVd-E1 (provider sample ID 37)	PSTVd-P1 (provider sample ID 40)
PSTVd-E2 (provider sample ID 38)	PSTVd-P2 (provider sample ID 41)
PSTVd-E3 (provider sample ID 39)	PSTVd-P3 (provider sample ID 42)

Sequence comparison result

Bidirectional sequencing provided complementary coverage across the full-length 359-nt PSTVd sequence. No nucleotide differences were detected at the consensus-sequence level between the PSTVd-E and PSTVd-P samples, and the consensus sequence of each sample was concordant with the inoculated PSTVd-S sequence (GenBank accession X58388.1).

Consensus calls were determined from the combined bidirectional chromatograms; isolated ambiguous single-direction base calls were resolved by inspection of the corresponding opposite-direction trace.

The original Sanger chromatograms are reproduced unmodified after the sequence alignment. The provider labels [227] and [228] on those chromatograms correspond to PSTVd-Seq1 and PSTVd-Seq2, respectively.

Note: Direct Sanger sequencing primarily reflects the predominant sequence population and does not exclude low-frequency sequence variants.

Consensus sequence alignment

The inoculated PSTVd-S sequence (X58388.1; 359 nt) is shown as the reference. Dots indicate identity with the reference sequence. The two sequencing directions provided complementary coverage of the full-length 359-nt PSTVd sequence and overlapped across most of the amplicon. Sequences are shown in the orientation of the X58388.1 reference sequence.

```
X58388.1      1 CGGAACTAAACTCGTGGTTCCTGTGGTTCACACCTGACCTCCTGACAAGAAAAGAAAAAA 60
PSTVd-E1        ............................................................
PSTVd-E2        ............................................................
PSTVd-E3        ............................................................
PSTVd-P1        ............................................................
PSTVd-P2        ............................................................
PSTVd-P3        ............................................................

X58388.1     61 GAAGGCGGCTCGGAGGAGCGCTTCAGGGATCCCCGGGGAAACCTGGAGCGAACTGGCAAA 120
PSTVd-E1        ............................................................
PSTVd-E2        ............................................................
PSTVd-E3        ............................................................
PSTVd-P1        ............................................................
PSTVd-P2        ............................................................
PSTVd-P3        ............................................................

X58388.1    121 AAAGGACGGTGGGGAGTGCCCAGCGGCCGACAGGAGTAATTCCCGCCGAAACAGGGTTTT 180
PSTVd-E1        ............................................................
PSTVd-E2        ............................................................
PSTVd-E3        ............................................................
PSTVd-P1        ............................................................
PSTVd-P2        ............................................................
PSTVd-P3        ............................................................

X58388.1    181 CACCCTTCCTTTCTTCGGGTGTCCTTCCTCGCGCCCGCAGGACCACCCCTCGCCCCCTTT 240
PSTVd-E1        ............................................................
PSTVd-E2        ............................................................
PSTVd-E3        ............................................................
PSTVd-P1        ............................................................
PSTVd-P2        ............................................................
PSTVd-P3        ............................................................

X58388.1    241 GCGCTGTCGCTTCGGCTACTACCCGGTGGAAACAACTGAAGCTCCCGAGAACCGCTTTTT 300
PSTVd-E1        ............................................................
PSTVd-E2        ............................................................
PSTVd-E3        ............................................................
PSTVd-P1        ............................................................
PSTVd-P2        ............................................................
PSTVd-P3        ............................................................

X58388.1    301 CTCTATCTAACTTGTTCCGGGGCGAGGGTGTTTAGCCCTTGGAACCGCAGTTGGTTCCT 359
PSTVd-E1        ...........................................................
PSTVd-E2        ...........................................................
PSTVd-E3        ...........................................................
PSTVd-P1        ...........................................................
PSTVd-P2        ...........................................................
PSTVd-P3        ...........................................................
```

No region-specific consensus nucleotide substitution was detected among PSTVd-E1-E3 and PSTVd-P1-P3.

File: 0001_32926082900361_(37)_[227].ab1 Run Ended: 2026-08-30 09:47:12 Signal G:934 A:1055 T:933 C:1487

Sample: 72901659534_32926082900361_(37)_[227]_PW Lane: 4 Base spacing: 14.12 339 bases in 4218 scans Page 1 of 1

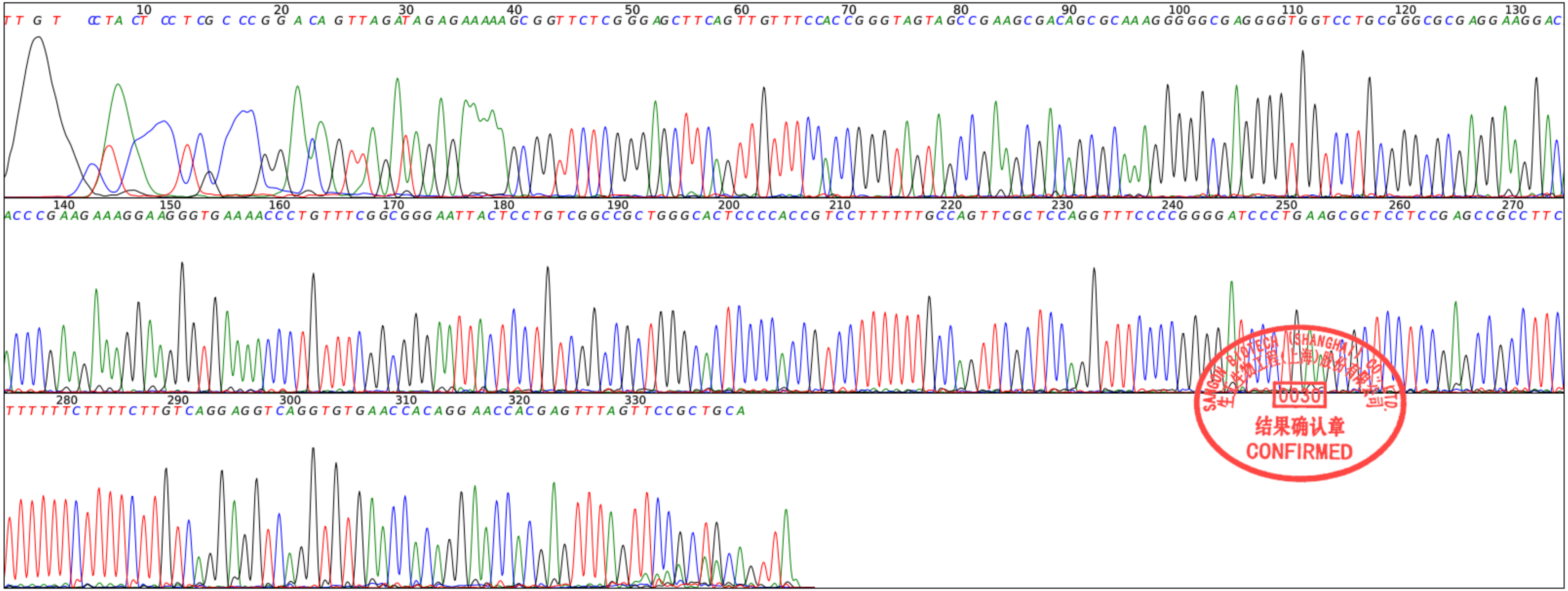

File: 0002_32926082900361_(37)_[228].ab1 Run Ended: 2026-08-31 18:55:10 Signal G:927 A:923 T:766 C:1129

Sample: 72901660170_32926082900361_(37)_[228]_PW Lane: 4 Base spacing: 14.19 351 bases in 4342 scans

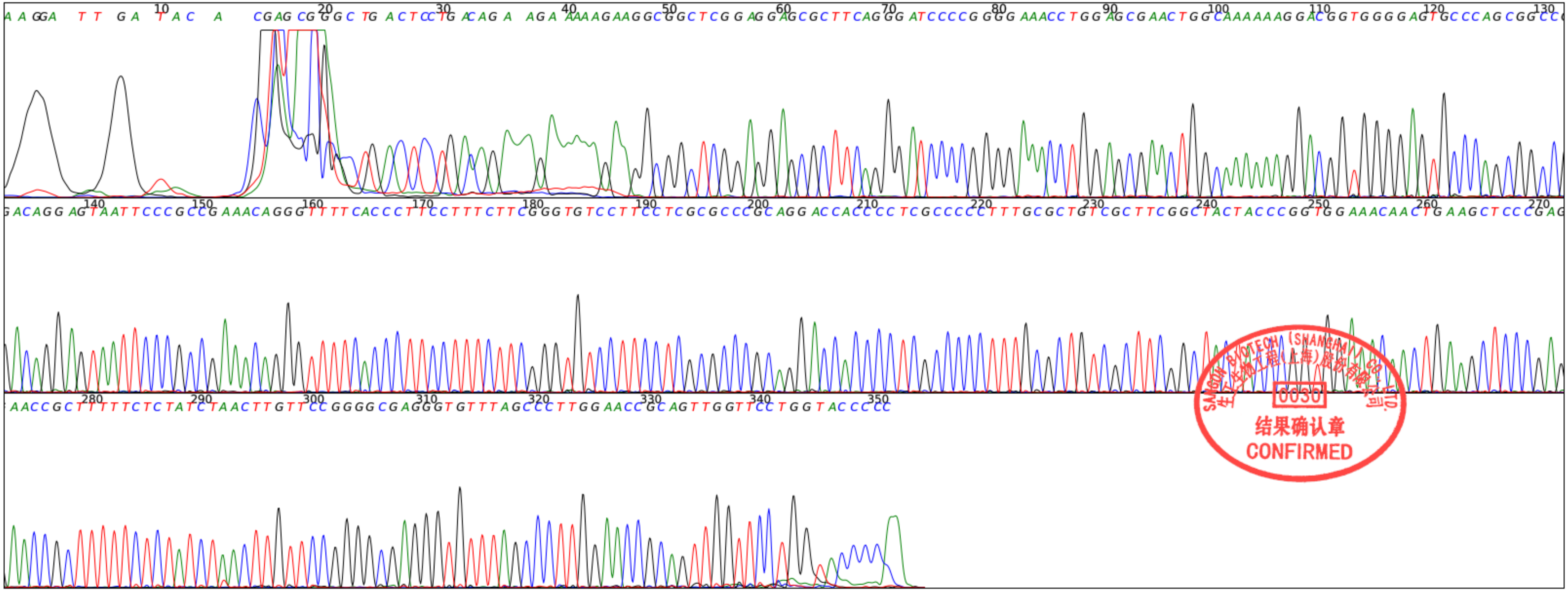

File: 0003_32926082900362_(38)_[227].ab1 Run Ended: 2026-08-30 09:47:12 Signal G:1199 A:1557 T:1297 C:2227

Sample: 72901659535_32926082900362_(38)_[227]_PW Lane: 4 Base spacing: 14.06 333 bases in 4218 scans Page 1 of 1

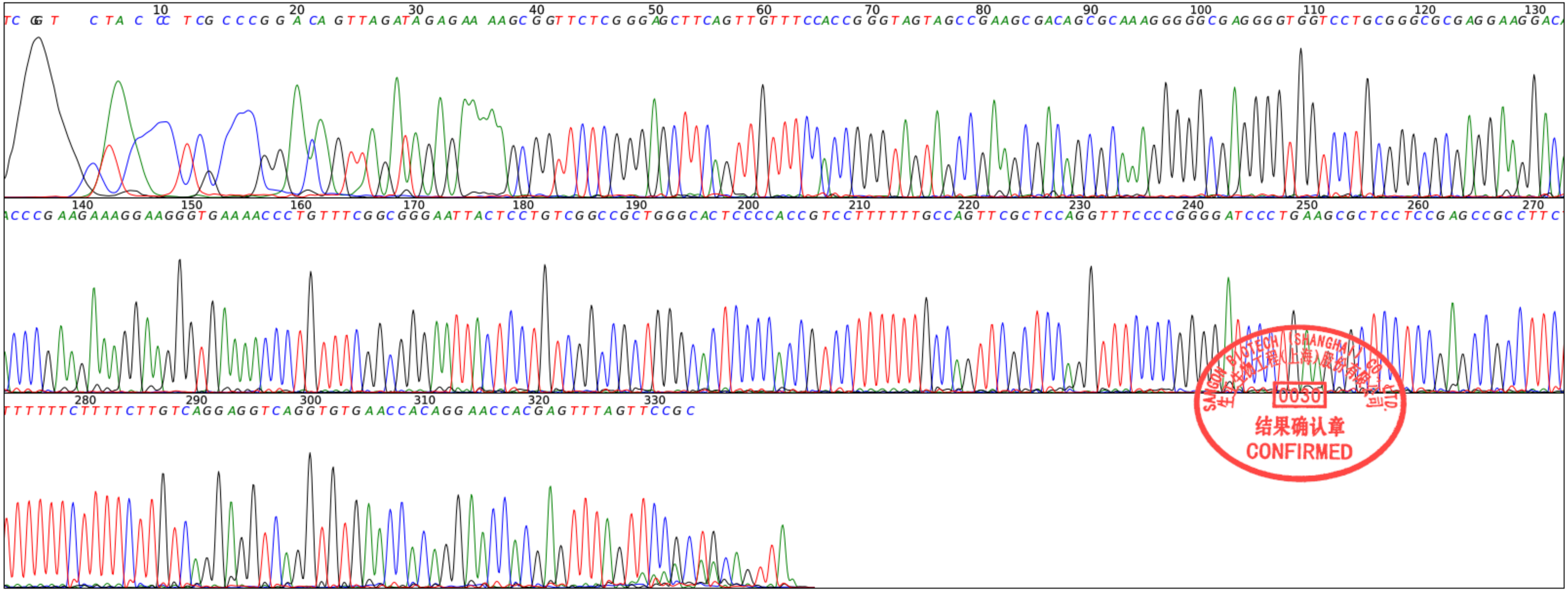

File: 0004_32926082900362_(38)_[228].ab1 Run Ended: 2026-08-31 18:55:10 Signal G:1345 A:1563 T:1236 C:1881

Sample: 72901660171_32926082900362_(38)_[228]_PW Lane: 4 Base spacing: 14.11 342 bases in 4206 scans Page 1 of 1

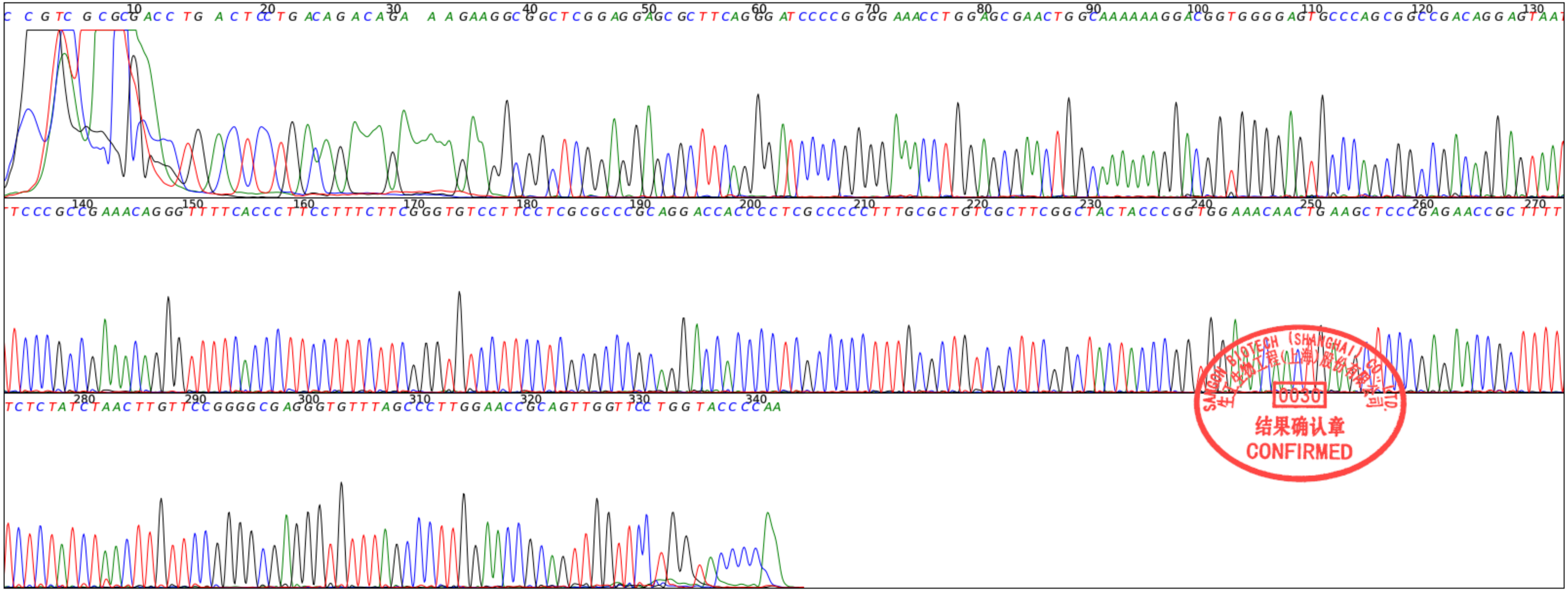

File: 0005_32926082900363_(39)_[227].ab1 Run Ended: 2026-08-30 09:47:12 Signal G:1362 A:1973 T:1679 C:2886

Sample: 72901659536_32926082900363_(39)_[227]_PW Lane: 4 Base spacing: 14.07 335 bases in 4213 scans Page 1 of 1

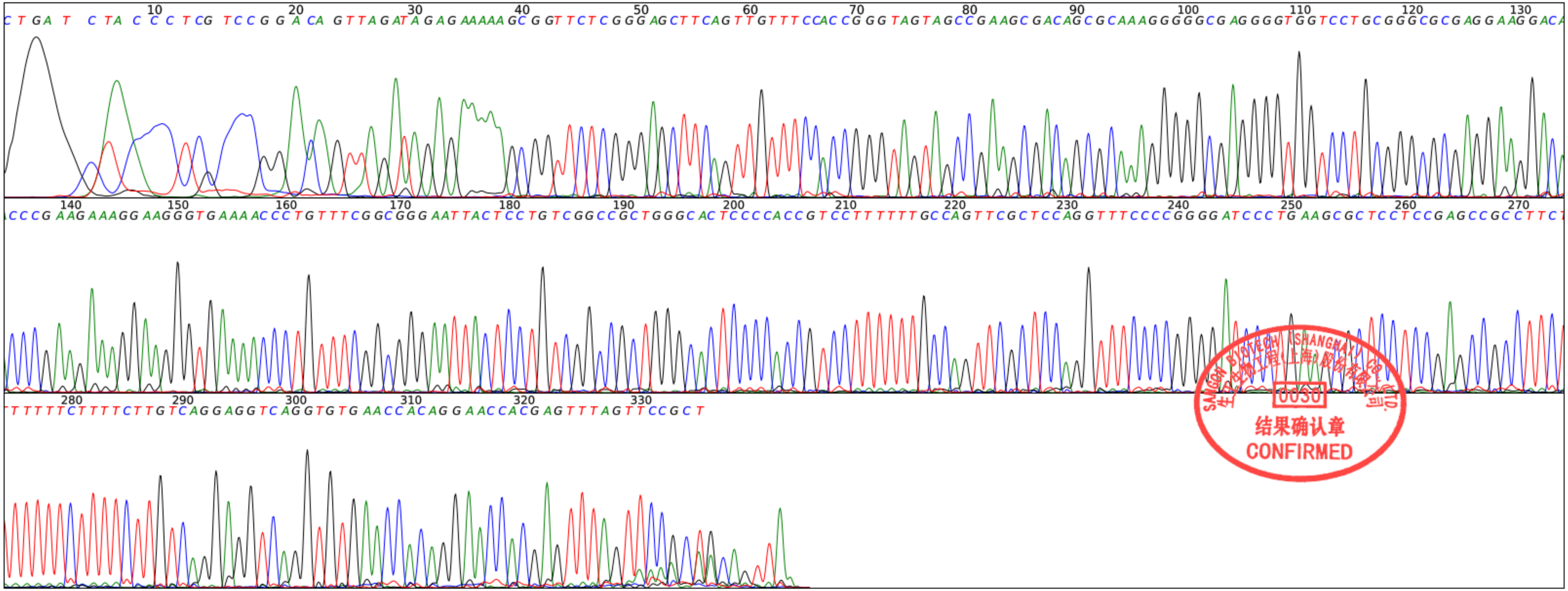

File: 0006_32926082900363_(39)_[228].ab1 Run Ended: 2026-08-31 18:55:10 Signal G:979 A:1314 T:1067 C:1471

Sample: 72901660172_32926082900363_(39)_[228]_PW Lane: 4 Base spacing: 14.06 336 bases in 4285 scans

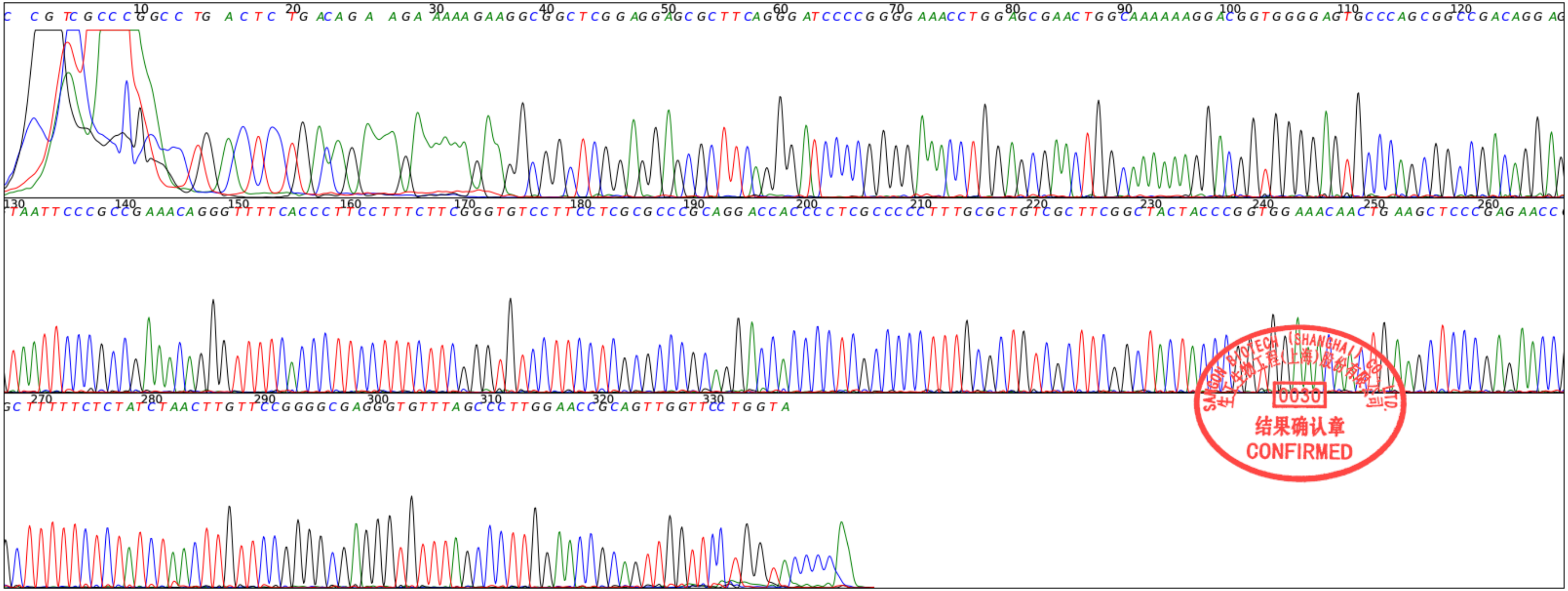

File: 0007_32926082900364_(40)_[227].ab1 Run Ended: 2026-08-30 09:47:12 Signal G:1379 A:1936 T:1784 C:2669

Sample: 72901659537_32926082900364_(40)_[227]_PW Lane: 4 Base spacing: 13.96 335 bases in 4222 scans Page 1 of 1

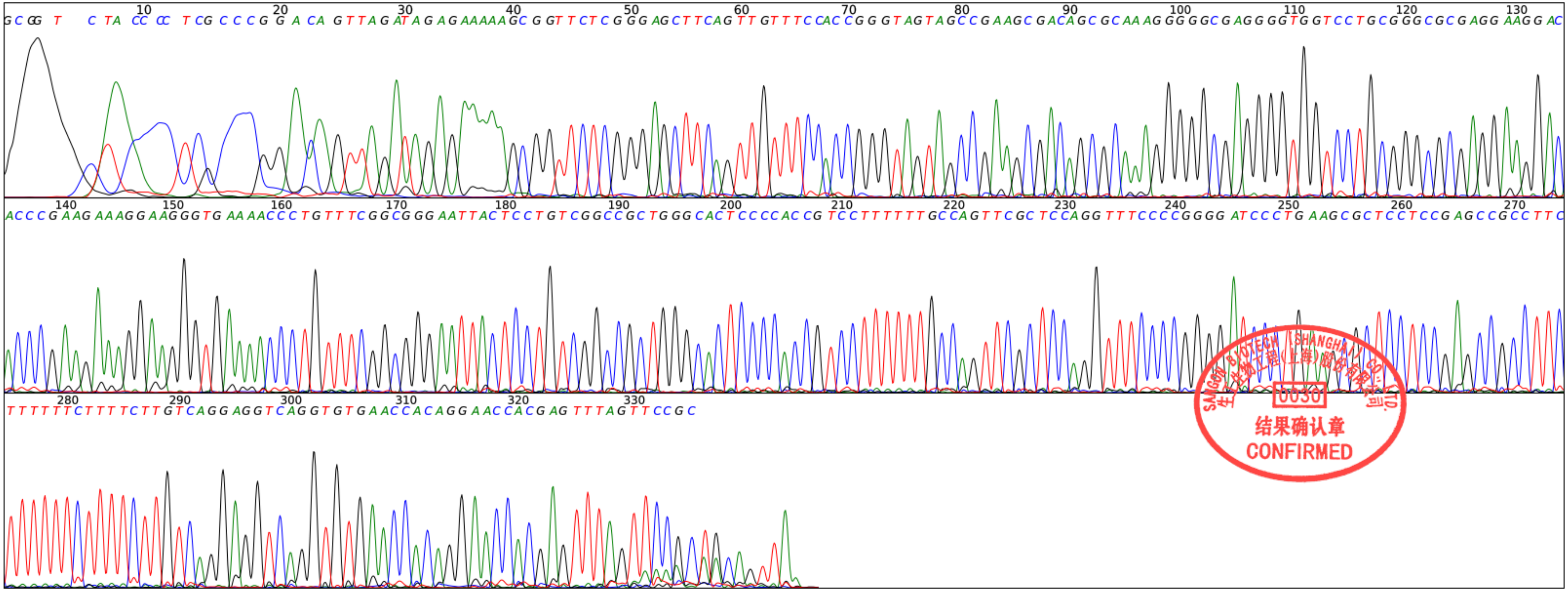

File: 0008_32926082900364_(40)_[228].ab1 Run Ended: 2026-08-31 18:55:10 Signal G:629 A:772 T:618 C:879

Sample: 72901660173_32926082900364_(40)_[228]_PW Lane: 4 Base spacing: 14.21 341 bases in 4280 scans Page 1 of 1

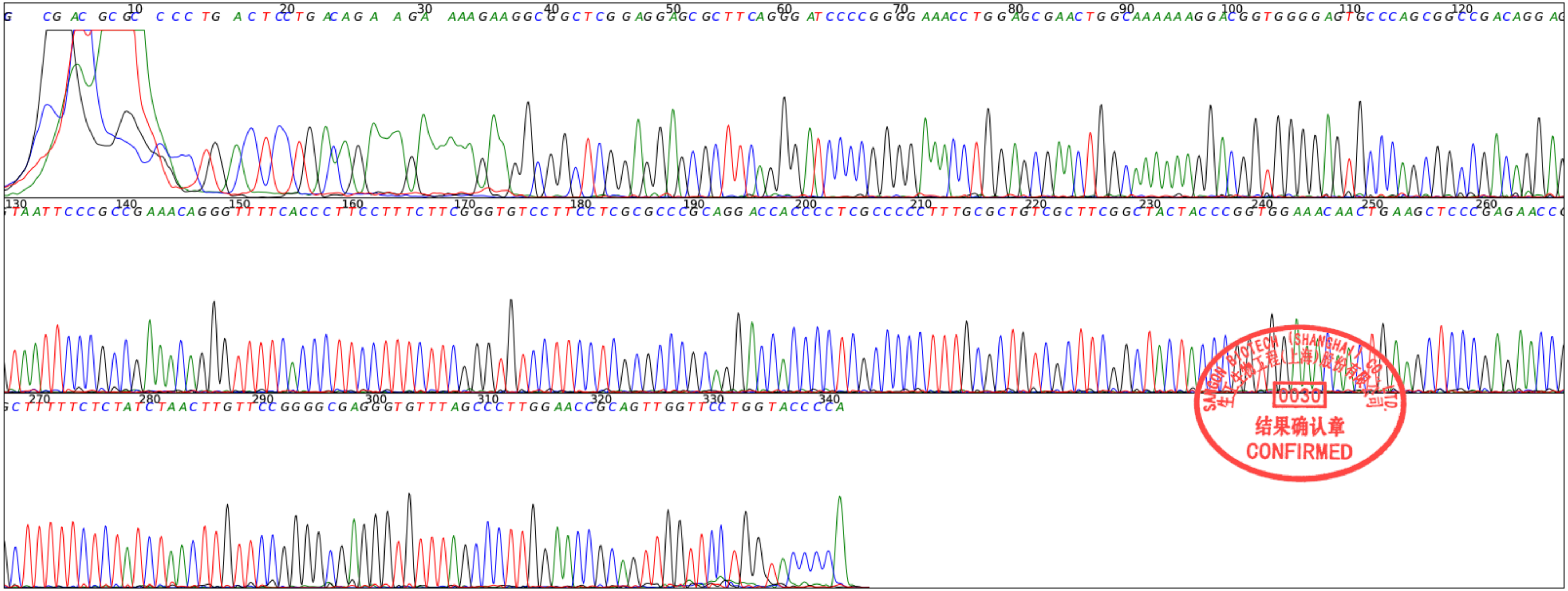

File: 0009_32926082900365_(41)_[227].ab1 Run Ended: 2026-08-30 09:47:12 Signal G:1362 A:1770 T:1482 C:2331

Sample: 72901659538_32926082900365_(41)_[227]_PW Lane: 4 Base spacing: 14.01 336 bases in 4219 scans Page 1 of 1

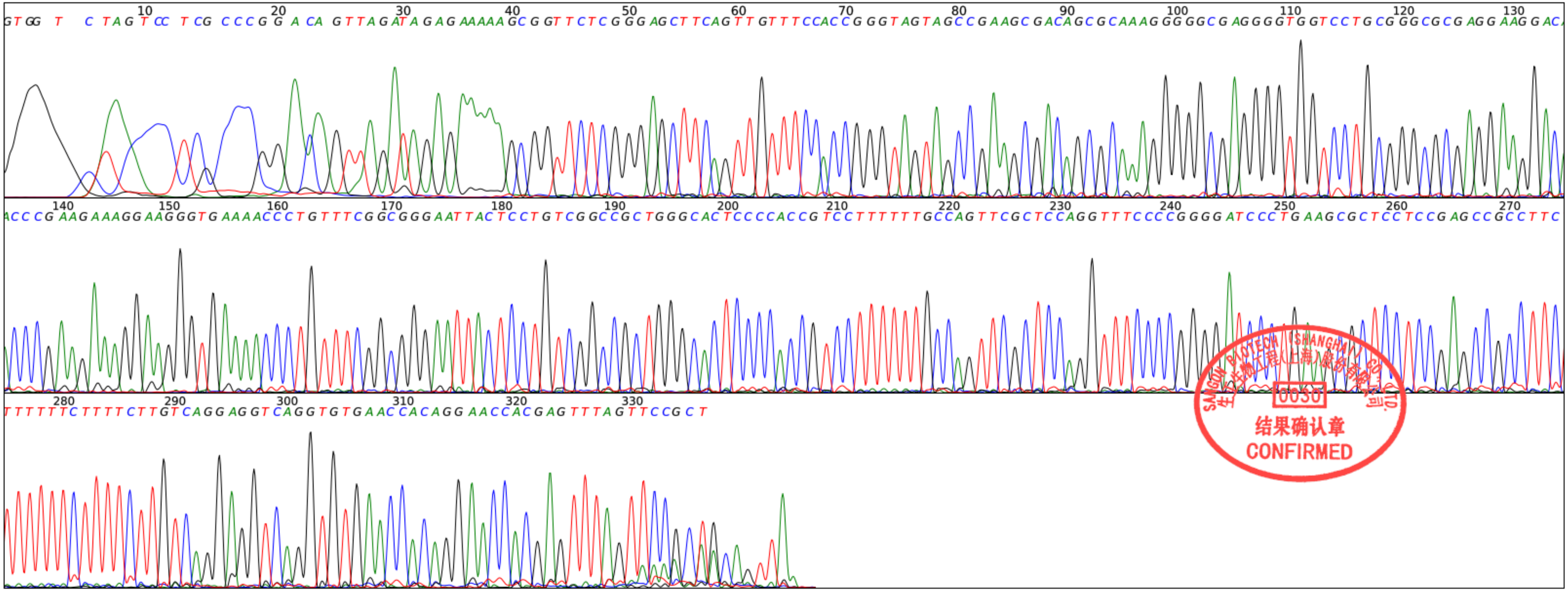

File: 0010_32926082900365_(41)_[228].ab1 Run Ended: 2026-08-31 18:55:10 Signal G:1564 A:1816 T:1446 C:2394

Sample: 72901660174_32926082900365_(41)_[228]_PW Lane: 4 Base spacing: 14.25 339 bases in 4277 scans Page 1 of 1

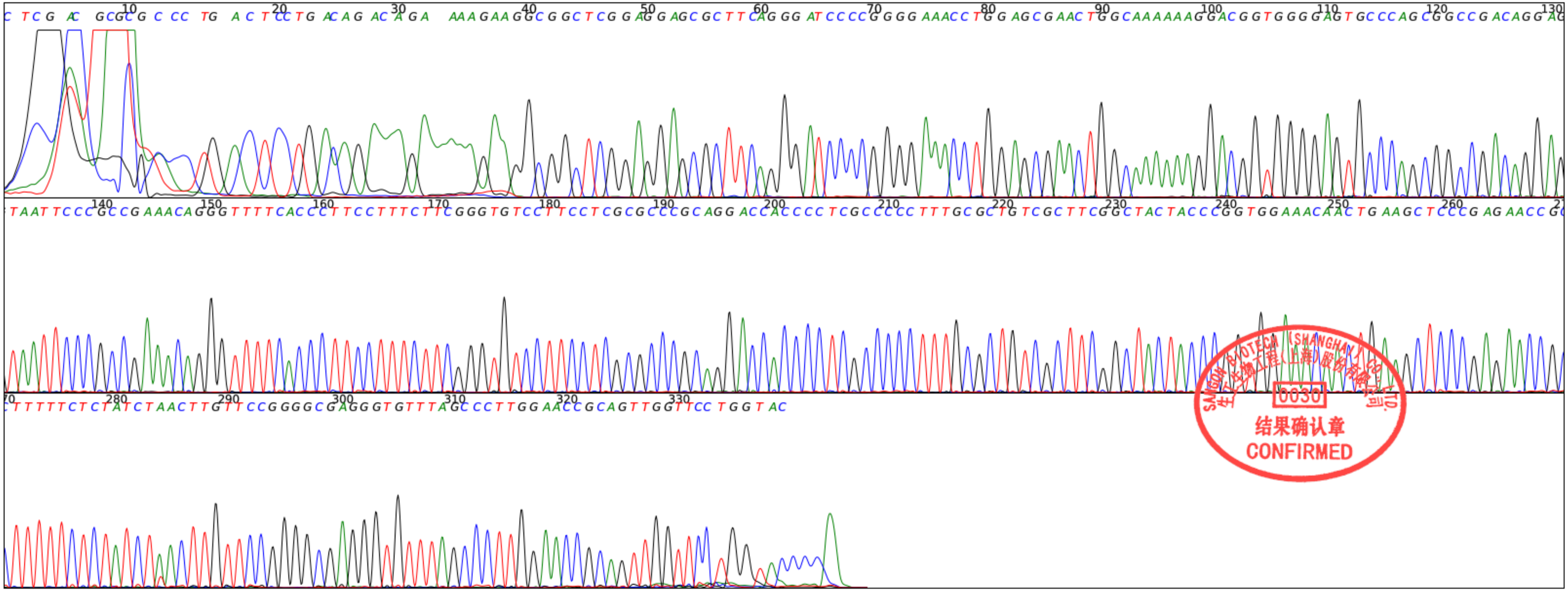

File: 0011_32926082900366_(42)_[227].ab1 Run Ended: 2026-08-30 09:47:12 Signal G:1058 A:1346 T:1166 C:1830

Sample: 72901659539_32926082900366_(42)_[227]_PW Lane: 4 Base spacing: 14.09 336 bases in 4216 scans

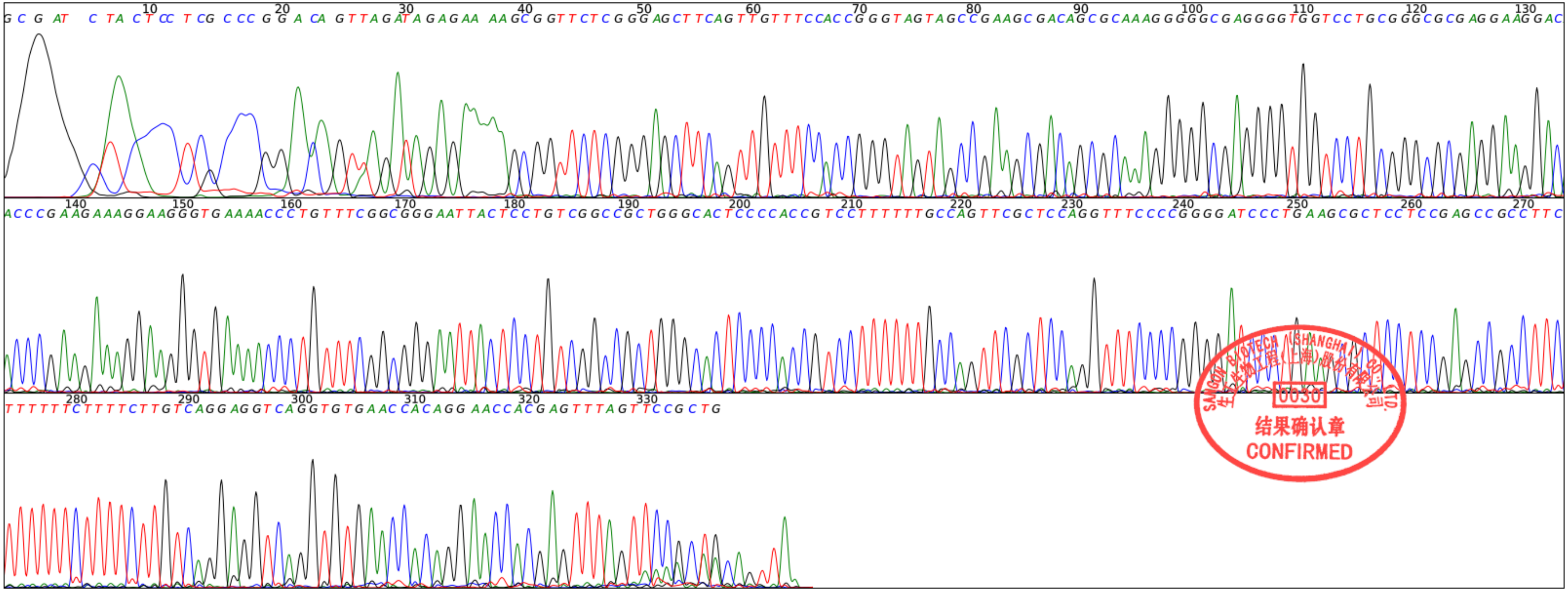

File: 0012_32926082900366_(42)_[228].ab1 Run Ended: 2026-08-31 18:55:10 Signal G:1253 A:1464 T:1162 C:1753

Sample: 72901660175_32926082900366_(42)_[228]_PW Lane: 4 Base spacing: 14.23 344 bases in 4270 scans Page 1 of 1

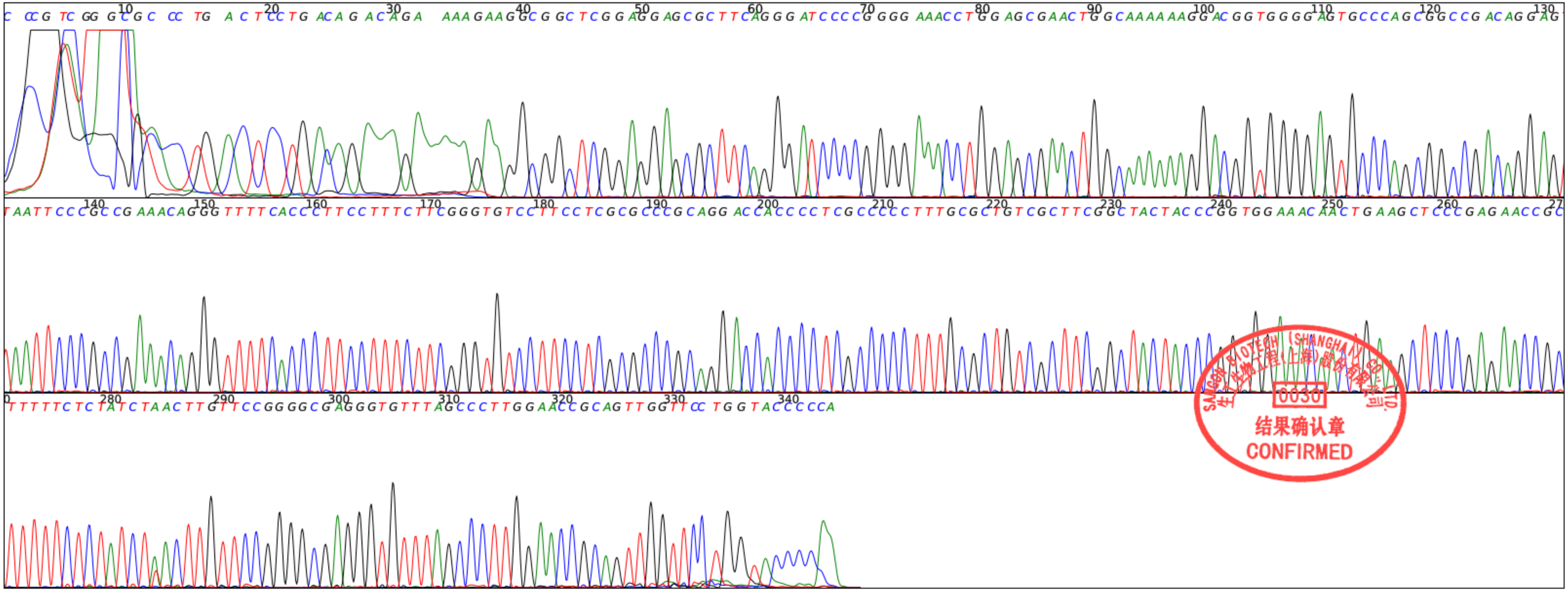

Supplement: Supplementary file 1 [file plants-15-02842-s001.zip › Supplementary File S1.pdf]
